# Tumor Treating Fields Suppression of Ciliogenesis Enhances Temozolomide Toxicity

**DOI:** 10.3389/fonc.2022.837589

**Published:** 2022-03-11

**Authors:** Ping Shi, Jia Tian, Brittany S. Ulm, Julianne C. Mallinger, Habibeh Khoshbouei, Loic P. Deleyrolle, Matthew R. Sarkisian

**Affiliations:** ^1^ Department of Neuroscience, University of Florida College of Medicine, Gainesville, FL, United States; ^2^ Department of Neurosurgery, University of Florida College of Medicine, Gainesville, FL, United States; ^3^ Preston A. Wells Jr. Center for Brain Tumor Therapy, University of Florida College of Medicine, Gainesville, FL, United States

**Keywords:** cilium, electrical fields, chemotherapy, brain tumor, ARL13B, treatment order

## Abstract

Tumor Treating Fields (TTFields) are low-intensity, alternating intermediate-frequency (200 kHz) electrical fields that extend survival of glioblastoma patients receiving maintenance temozolomide (TMZ) chemotherapy. How TTFields exert efficacy on cancer over normal cells or interact with TMZ is unclear. Primary cilia are microtubule-based organelles triggered by extracellular ligands, mechanical and electrical field stimulation and are capable of promoting cancer growth and TMZ chemoresistance. We found in both low- and high-grade patient glioma cell lines that TTFields ablated cilia within 24 h. Halting TTFields treatment led to recovered frequencies of elongated cilia. Cilia on normal primary astrocytes, neurons, and multiciliated/ependymal cells were less affected by TTFields. The TTFields-mediated loss of glioma cilia was partially rescued by chloroquine pretreatment, suggesting the effect is in part due to autophagy activation. We also observed death of ciliated cells during TTFields by live imaging. Notably, TMZ and TTFields have opposing effects on glioma ciliogenesis. TMZ-induced stimulation of ciliogenesis in both adherent cells and gliomaspheres was blocked by TTFields. Surprisingly, the inhibitory effects of TTFields and TMZ on tumor cell recurrence are linked to the relative timing of TMZ exposure to TTFields and ARL13B^+^ cilia. Finally, TTFields disrupted cilia in patient tumors treated *ex vivo*. Our findings suggest that the efficacy of TTFields may depend on the degree of tumor ciliogenesis and relative timing of TMZ treatment.

## Introduction

High-grade gliomas in adult, such as glioblastoma (GBM), usually have dismal prognoses due to the resistance and recurrence following all standard of care treatments. These treatments include a combination of surgical resection (if possible), irradiation, and temozolomide (TMZ) chemotherapy, the combination of which extends survival only for a few months (1, 2), indicating novel treatments are urgently needed. One of the latest Food and Drug Administration-approved treatments for GBM patients is Tumor Treating Fields (TTFields) (Optune®), a device/electrode set that patients wear during their treatment that delivers low-intensity (1–3 V/cm), alternating intermediate-frequency (200 kHz) electric fields across the head. Combining maintenance treatment with TMZ, TTFields significantly increases overall survival several months beyond TMZ alone (3, 4). Thus, TTFields are considered a new standard of care option (5). However, our understanding of how TTFields differentially targets gliomas over normal cells, interacts or enhances current therapies, or whether tumor cell characteristics predict sensitivity to TTFields remain unanswered questions.

The antitumor effects of TTFields do not occur *via* a single mechanism of action but rather a variety of cellular and molecular alterations [for review, see; (6)]. For example, TTFields disrupt the microtubular organization of mitotic spindle affecting normal cytokinesis and mitosis (7–9), as well as suppressing cell migration and invasion (10, 11). TTFields inhibit DNA damage repair and induce replication stress (12, 13). TTFields can induce autophagy (14) and promote immunogenic cell death (15). TTFields also change the cell plasma membrane permeability to a greater extent in tumor cells compared with primary dermal fibroblasts (16). Such membrane changes may be linked to calcium channel activation and rapid calcium influx that occurs during TTFields (17). Altogether, these factors may cumulatively result in reduced proliferative and invasive capacity of glioma cells and enhanced sensitization to current therapies.

Many pathways impacted by TTFields are regulated by or involve signaling linked to the primary cilium [for review, see; (18–20)]. Primary cilia are nonmotile, microtubule-based organelles extending from the mother centriole of the basal body. Cilia must be disassembled so centrioles within the basal body can duplicate, segregate, and be repurposed for mitosis. Cilia are ensheathed by plasma membrane distinct from the membrane of the cell body (21–24) and generally depend on intraflagellar transport machinery for their outgrowth which mobilizes cargo anterogradely to the ciliary tip and retrogradely back to the cell body (25). At any given time, cilia are present in up to 30% or more of high-grade glioma cells (26). Nothing is known about how electrical field stimulation impact primary cilia on glioma cells. In human adipose-derived stem cells, brief exposures (4 h/day) to low-intensity (1 V/cm), low-frequency (1 Hz) nonalternating electric fields were reported to induce osteogenesis *via* primary cilia (27). Electrical field-induced osteogenic responses were absent when ciliogenesis was inhibited using siRNA targeting an essential ciliogenesis gene intraflagellar transport 88 (*IFT88*) (27). Exposure to 16 Hz pulsed electromagnetic fields protected ciliary morphology against cigarette-smoke-induced damage in osteoprogenitor cells (28). Thus, whatever role(s) primary cilia serve on glioma cells, they may be sensitive to or stimulated by the much higher therapeutic frequencies used in TTFields therapy.

The cilia on glioma cells may play a role in resistance to TMZ (29, 30). For example, cilia depletion mediated by CRISPR/Cas9 depletion of *PCM1* or *KIF3a*, two critical ciliogenesis genes, sensitized GBM cells to TMZ (29). More recently, TMZ was shown to induce enhancer of zeste homologue 2 (EZH2) which targets the expression of ADP ribosylation factor 13b (ARL13B), a regulatory GTPase highly concentrated in glioma cilia (26, 31), as an adaptive mechanism that promotes chemoresistance (30). Knockdown of ARL13B/cilia using shRNA in patient-derived xenografts *in vivo* not only slowed tumor growth but increased sensitivity to TMZ *in vivo*. Thus, if TTFields affects ARL13B or ARL13B^+^ cilia, the sensitivity of glioma cells to TMZ could be enhanced. The goals of this study were to determine whether and how TTFields at the clinical frequency (200 kHz) affects glioma ciliogenesis compared with normal neural cell types *in vitro*. We also examined how TMZ alone versus TMZ plus TTFields affects ciliogenesis and proliferation on both ARL13B^+^ (ciliated) and ARL13B^−^ (nonciliated) glioma cell lines characterized previously (32). Finally, we explored whether TTFields affects ARL13B^+^ cilia in the patient tumor microenvironment.

## Materials and Methods

### Cell Culture

L0 (grade IV glioblastoma from a 43-year-old man) and S7 (grade II glioma from a 54-year-old woman with EGFR amplification) cell lines were isolated and maintained as previously described (26, 33–35). *ARL13B*-deficient S7 cells were generated using CRISPR/Cas9 as previously described (32). L0 and S7 cells were grown as floating spheres and maintained in NeuroCult NS-A proliferation medium and 10% proliferation supplement (Cat# 05750 and #05753; STEMCELL Technologies, Vancouver, Canada), 1% penicillin–streptomycin (Cat# 15140122; ThermoFisher, Waltham, MA, USA), 20 ng/ml human epidermal growth factor (hEGF) (Cat# 78006; STEMCELL Technologies, Vancouver, Canada), and 10 ng/ml basic fibroblast growth factor (bFGF) (Cat# 78003; STEMCELL Technologies, Vancouver, Canada). For S7 cells, the media were supplemented with 2 μg/ml heparin (Cat# 07980; STEMCELL Technologies, Vancouver, Canada). All cells were grown in a humidified incubator at 37°C with 5% CO_2_. When cells reached confluency, or spheres reached approximately 150 μm in diameter, they were enzymatically dissociated by digestion with Accumax (Cat# AM-105; Innovative Cell Technologies, San Diego, CA, USA) for 10 min at 37°C. For human cells grown on glass coverslips, NeuroCult NS-A Proliferation medium was supplemented with 10% heat-inactivated fetal bovine serum (FBS) (Cat# SH30070.03HI; Cytiva, Marlborough, MA, USA).

Primary neural cultures were similar to previously described (32, 36). Briefly, acutely microdissected C57/BL6 mouse cortices from postnatal day 0 to 2 pups were dissected into Gey’s Balanced Salt Solution (Cat# G9779; Sigma-Aldrich, St. Louis, MO, USA) at ~37°C under oxygenation for ~20 min. Dissociated cells were triturated with pipettes of decreasing bore size, pelleted by centrifugation at 1,500 rpm for 3–5 min, and resuspended and plated in glial medium containing DMEM (Cat# SH3002201; Cytiva HyClone, Marlborough, MA, USA), FBS (Cat# 50-753-2981; Gemini BioProducts, West Sacramento, CA, USA), insulin (Cat# 15500; Sigma-Aldrich, St. Louis, MO, USA), Glutamax (Cat# 35050061; Gibco, Waltham, MA, USA), and penicillin–streptomycin (Cat# 15140122; Gibco, Waltham, MA, USA). Cells were plated at a density of 80,000 cells/coverslip on 12-mm glass coverslips coated with 0.1 mg/ml poly-d-lysine followed by 5 μg/ml laminin in minimal essential medium. After approximately 2 h, cells were supplemented with 2 ml neuronal media containing Neurobasal A (Cat# 10888022; Gibco) supplemented with B27 (Cat# A3582801; Gibco), GlutaMAX (35050061; Gibco), kynurenic acid (Sigma-Aldrich, Cat# K3375), and glial cell line-derived neurotrophic factor (GDNF; Cat# SRP3200; Sigma-Aldrich). Every 4 days, half of the media was replaced with fresh neuronal media as described above but lacking kynurenic acid and GDNF. On DIV12, coverslips were transferred into TTFields dishes and fixed after 24 h or 3 days after treatment as described below.

### TTFields Induction and Timelapse Imaging

For adherent and spheres, 5 × 10^4^ cells were seeded in 2 ml growth media with or without 10% FBS, respectively. Adherent cells, spheres, or biopsies were placed in TTFields ceramic dishes, each dish approximately the size of a single well of a 6-well plate, and mounted into Inovitro™ base plates (Novocure Ltd., Haifa, Israel). The base plates were connected to a power generator which delivered TTFields at a frequency of 200 kHz at a target intensity of 1.62 V/cm (37). During TTFields treatment, cells were maintained in an incubator (ESCO Technologies, Horsham, PA, USA) with the ambient temperature set to 18°C with 5% CO_2_ and a target temperature of 37°C inside each ceramic dish. Treatment duration is as indicated but ranged from 1 to 72 h for a single treatment. To prevent media evaporation during TTFields application, parafilm was placed over each TTFields ceramic dish. In between repeated exposures or for recovery experiments, cells were dissociated and transferred back to a regular incubator. Control samples were grown at 37°C in 5% CO_2_ in 6-well plates. In some experiments, we pretreated cells before TTFields with either vehicle or specified drugs. Unless otherwise stated, data in each experiment were pooled from at least 4 dishes per condition and per timepoint.

For timelapse imaging combined with TTFields, we plated 50,000 cells in S7/L0 media supplemented with 5% FBS into 35 mm glass bottom culture dishes (Cat# 81158; Ibidi, Gräfelfing, Germany) which were maintained at 37°C in 5% CO_2_. Twenty-four hours before imaging at about 70% confluency, cells were transfected with 500 ng total cDNA/dish of pDest-Arl13b:GFP (gift from T Caspary) and pCMV-myc/mCherry:hOFD1 (Vectorbuilder.com, vector ID: VB201119-1128fyp) using Lipofectamine 3000 (Cat# L3000015; Life Technologies, Carlsbad, CA, USA). A TTFields-delivering ceramic insert was placed into the culture dish and connected to a generator that delivered TTFields at a frequency of 200 kHz at an approximate intensity of 1.2 V/cm and target temperature of 37°C inside each dish. Imaging was conducted on an inverted Zeiss AxioObserver D1 microscope using a Zeiss 40×/0.95 plan Apochromat air objective. The microscope stage was equipped with a Tokai Hit stage incubation system that maintained a humid environment and ambient temperature of 22°C–23°C and 5% CO_2_. Baseline images were captured every minute, whereas after TTFields onset, images were collected every 5 min with exposure times ranging in duration from 400 to 750 ms (EGFP) and 300–400 ms (Cy3) per image. Image acquisition and processing were performed using Zeiss ZEN software.

### *Ex Vivo* Culture and TTFields Treatment

In accordance with our institutional IRB protocol (# 201902489), we collected several fresh, surgically resected tumor biopsies that were subsequently pathologically confirmed. Within 1 h of the resection, biopsies were taken to the laboratory and dissected into several pieces using a sterile scalpel blade. Tissues were immediately fixed and/or transferred into 2 ml of S7 media for culture at 37°C in 5% CO_2_ or transferred into TTFields dishes for a 24-h exposure as described above. Following TTFields, control and treated samples were fixed and prepared as described above.

### Cell Growth, Viability Assays

For cell proliferation assay, cells (2.5–5 × 10^4^) were seeded in 2 ml of growth media per well in 6-well plates or in 1 ml of growth media per well in 24-well plates for indicated duration. Cells were then treated with various drugs including chloroquine (CQ) (Cat# C6628; Sigma) (20 µM diluted in sterile water), TMZ (Cat# T2577; Sigma) (0.3 to 100 µM diluted in DMSO), 1,2-bis(o-aminophenoxy)ethane-*N*,*N*,*N*′,*N*′-tetraacetic acid (BAPTA) (Cat# B1205; Sigma) (1 µM diluted in DMSO), or ethylene glycol-bis(β-aminoethyl ether)-*N*,*N*,*N*′,*N*′-tetraacetic acid (EGTA) (Cat# RES3010E; Sigma) (0.6 mM diluted in DMSO). After indicated treatment durations, cells were enzymatically dissociated and replaced in ×1 phosphate-buffered saline (PBS). Total cell counts were collected using a Bio-Rad TC20 automated cell counter. Bar graphs show the mean (+/− SEM) and were analyzed statistically using analysis of variance (ANOVA).

### Immunostaining

For immunocytochemical (ICC) and immunohistochemical (IHC) analyses, samples were fixed at indicated timepoints with 4% paraformaldehyde in 0.1 M phosphate buffer (4% PFA) for 30 min or ice-cold methanol for 15 min (ICC) to 1 h (IHC) and washed with 1× PBS. Spheres or biopsies were cryoprotected in 30% sucrose in PBS followed by a 1:1:30% sucrose and optimal cutting temperature compound (OCT) (Cat# 4585; Fisher Healthcare, Loughborough, UK), frozen in OCT over liquid N_2_ and cryosection at 16 µm. Samples were stained for the indicated primary antibodies (**Table S1**). Samples were incubated in blocking solution containing 5% normal donkey serum (NDS) (Cat# NC9624464; Jackson Immunoresearch, West Grove, PA, USA) and 0.2% Triton-X 100 in 1× PBS for 1 h and then incubated in primary antibodies with 2.5% NDS and 0.1% Triton-X 100 in 1× PBS either for 2 h at room temperature (RT) or overnight at 4°C. For samples stained sequentially with mouse antibodies against gamma- and acetylated alpha-tubulin, samples were blocked with donkey anti-mouse IgG Fab fragments (20 μg/ml; Cat# 715-007-003; Jackson Immunoresearch, West Grove, PA, USA) as previously described (26). Appropriate FITC-, Cy3-, or Cy5-conjugated secondary antibodies (1:1,000; Jackson ImmunoResearch) in 2.5% NDS with 1× PBS were applied for 1–2 h at RT, and coverslips were mounted onto Superfrost™ Plus-coated glass slides (Cat# 12-550-15; Fisher Scientific, Waltham, MA, USA) in Prolong Gold antifade media containing DAPI (Cat# P36935; ThermoFisher). Stained coverslips were examined under epifluorescence using an inverted Zeiss AxioObserver D1 microscope using a Zeiss 40×/0.95 plan Apochromat air objective or a Zeiss 63×/1.4 plan Apochromat oil objective. Images were captured and analyzed using Zeiss ZEN software.

## Results

### Effects of Different Durations of TTFields Exposure on Low- and High-Grade Patient Glioma Cell Primary Ciliogenesis

**Figure 1:**
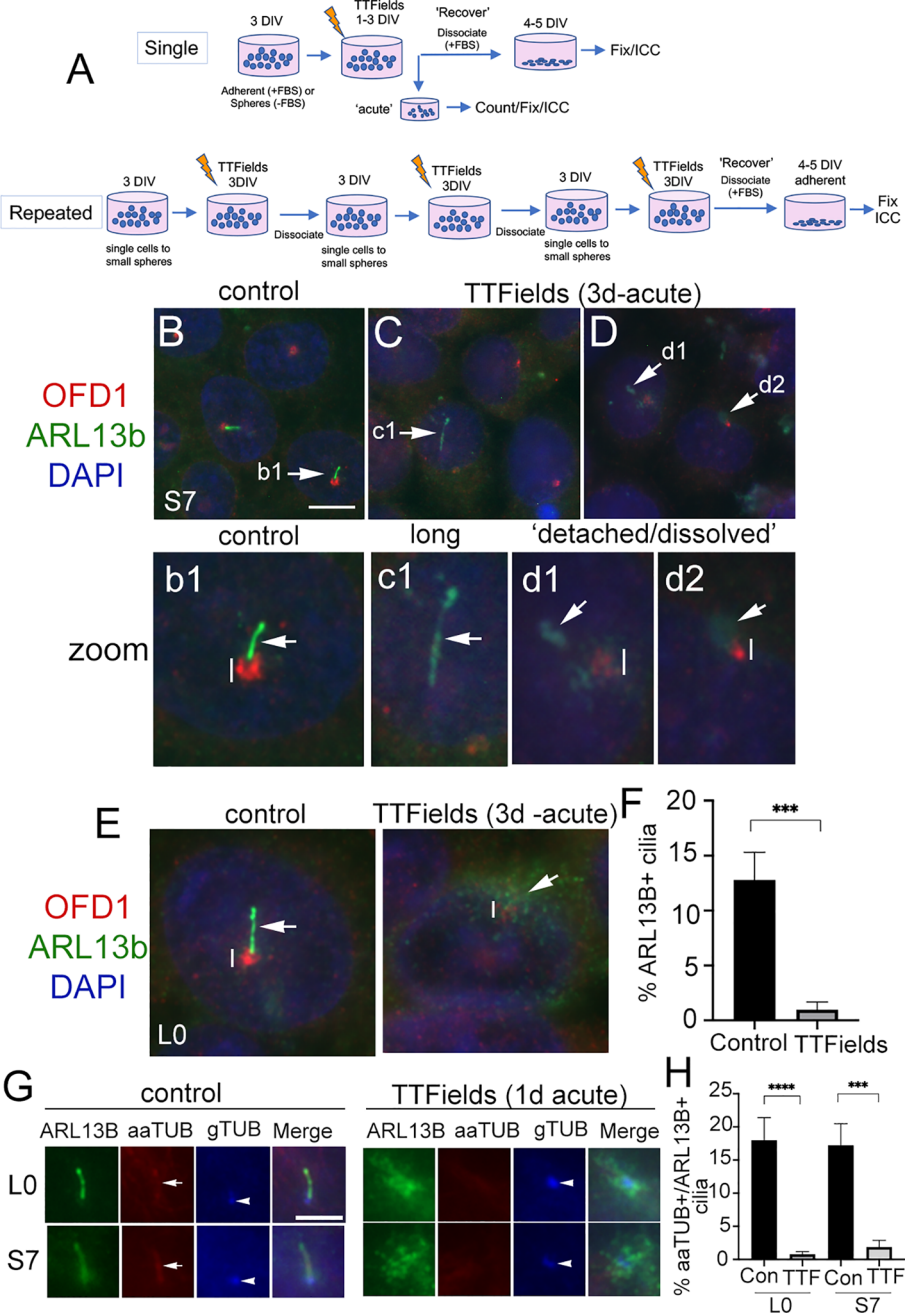
Primary cilia on patient-derived glioma cells are ablated by TTFields. **(A)** General approach to treat cells with a single or repeated exposure to TTFields *in vitro*. Cells that were grown adherent on glass coverslips had media supplemented with 10% fetal bovine serum (FBS). **(B–D)** S7 control cells stained for OFD1 (red), which clusters around basal bodies/centrioles, and ARL13B (green) which are enriched in the primary cilium. Nuclei are labeled with DAPI (blue). In control **(B)**, ARL13B^+^ cilia (arrow in **b1** zoom) from OFD1^+^ puncta (vertical line). In S7 TTFields-treated cells **(C, D)**, ARL13B^+^ cilia appeared elongated (e.g., **c1**), detached, or separated away from the OFD1^+^ basal body (e.g., **d1**) or somewhat dissolved or cloudy in appearance at OFD1 clusters (e.g., **d2**). OFD1 clusters in TTFields-treated cells appeared smaller/less intense compared with control (compare vertical lines between **b1** and **d1**/**d2**). **(E)** Control or 3 days of TTFields-treated L0 cells stained similar to **(B)**. Control cells display clear OFD1+ basal body (vertical line) at the base of ARL13B^+^ cilia (arrow). TTFields-treated cells displayed less intensely labeled OFD1^+^ basal body (vertical line) with surrounding dispersed/cloudy puncta of ARL13B (arrow). **(F)** Percent of ARL13B^+^ cilia in L0 cells in control vs. TTFields. **(G)** L0 (upper row) and S7 (bottom row) cells stained for ARL13B (green), acetylated alpha tubulin (aaTUB, red), and gamma-tubulin (gTUB, blue). Control cells show ARL13B+ cilia colocalized with aaTUB (arrow) with gTUB^+^ basal bodies (arrowheads). TTFields-treated cells have clustered/dispersed ARL13B signal with no clear aaTUB^+^ axoneme associated with the gTUB^+^ basal body. **(H)** Percent of aaTUB^+^/ARL13B^+^ cilia in control or TTFields-treated L0 and S7 cells. ^***^ *p* < 0.001, ^****^ *p* < 0.0001 (ANOVA). Scale bars (in µm) = 10 **(B)** and 5 **(G)**.

We exposed two patient-derived glioma cell lines, L0 (a grade IV glioblastoma) and S7 (a grade II glioma) that grow primary cilia (26, 29, 31, 32, 38) to TTFields. We used Novocure’s Inovitro™ system to deliver low-intensity (1–4 V/cm), 200 kHz alternating electric fields to cultured cells which presumably mimic the type of fields delivered by the Optune^®^ device in patients, similar to recent studies (16, 39). Generally, glioma cells were grown adherent (in serum) on coverslips or as free-floating spheres (without serum) for 3 days *in vitro* (DIV), then performed a single exposure to TTFields for up to 1 day or 3 continuous days at which point we analyzed cells immediately (“acute”) (**Figure 1A**). For repeated TTFields exposures, we dissociated cells after 3 days of continuous TTFields and repeated the cycle 2 more times (**Figure 1A**). For recovery, after the last day of single or repeated TTFields exposures, we dissociated spheres and cultured cells adherently (with serum) on coverslips and examined cells after 4–5 days.

After a single 3-day exposure to TTFields, we immunostained cells for ARL13B and orodigital facial syndrome 1 (OFD1), a protein that concentrates around the basal body (40–42). In control S7 cells, ARL13B+ cilia were readily identifiable extending from OFD1^+^ basal bodies (**Figures 1B** **, b1**). After TTFields, the presence of ARL13B^+^ cilia was largely undetected. Cilia that remained were typically elongated (**Figures 1C** **, c1**), or appeared detached from (**Figures 1D** **, d1**) or dissolved (**Figures 1D** **, d2**) around the basal bodies. Most TTFields-treated cilia displayed reduced intensity of OFD1 around the basal body compared with control (**Figures 1** **b1, d1, d2** and **Figure S1**). We observed a similar phenomenon in L0 cells (**Figure 1E** and **Figure S1**). The appearance (**Figure 1E**) and percent of ARL13B^+^ cilia (**Figure 1F**) in L0 cells were significantly reduced. The effects of TTFields on glioma cilia can be seen within 24 h posttreatment. In both L0 and S7 cell lines, there was a significant loss of ARL13B^+^ cilia after TTFields exposure (**Figures S2A–F**). To confirm that TTFields are affecting the cilium and not just ARL13B localization along the ciliary membrane, we performed triple immunostaining to label a different component of cilia axoneme, acetylated-alpha tubulin (aaTUB) along with gamma-tubulin (gTUB) a microtubule component of the basal body/centriole, and ARL13B. In both L0 and S7 control cells, we found cilia that colocalized aaTUB^+^ and ARL13B^+^ extended from gTUB^+^ basal bodies (**Figure 1G**). However, after TTFields exposure, ARL13B puncta clustered around gTUB^+^ basal body/centrioles without obvious aaTUB^+^ axoneme extending from gTUB^+^ puncta (**Figure 1G**). Quantification of cilia with both aaTUB^+^ and ARL13B^+^ cilia revealed significantly reduced frequencies after TTFields exposure in the two cell lines (**Figure 1H**), indicating that TTFields disrupt the integrity of the entire organelle.

The above observations suggest TTFields effects on the cilia of glioma initiate within hours. Indeed, TTFields have been shown to disrupt glioma cell membrane permeability within the first hour of treatment (16). Thus, we examined the cilia axoneme and membrane 1 and 6 h after TTFields using antibodies against aaTub, gTUB, ARL13B, and inositol polyphosphate-5-phosphatase E (INPP5e). INPP5e localizes to the ciliary membrane where it interacts with ARL13B (22, 43). We found that after 6 h, cilia appeared longer than controls in both cell lines. The elongated ARL13B^+^ cilia displayed underlying colocalization with aaTUB^+^, suggesting that TTFields may stimulate a transient lengthening of the entire organelle within hours (**Figures S3A, C**). We also observed some anomalies in the ciliary distribution of ARL13B and INPP5e staining. ARL13B and INPP5e seemed to distribute evenly along the ciliary axoneme in control, but after 60 min and 6 h exposures to TTFields, the staining pattern appear clustered or polarized toward the proximal and distal tips of the cilium (**Figures S3A–C**). In S7 cells, we also observed unusual clusters of INPP5e surrounding the basal body after TTFields exposure (**Figure S3B**). Thus, in addition to a ciliary lengthening that precedes the loss of cilia, TTFields affect properties of the cilia membrane and surrounding base within hours of exposure *in vitro*.

**Figure 2:**
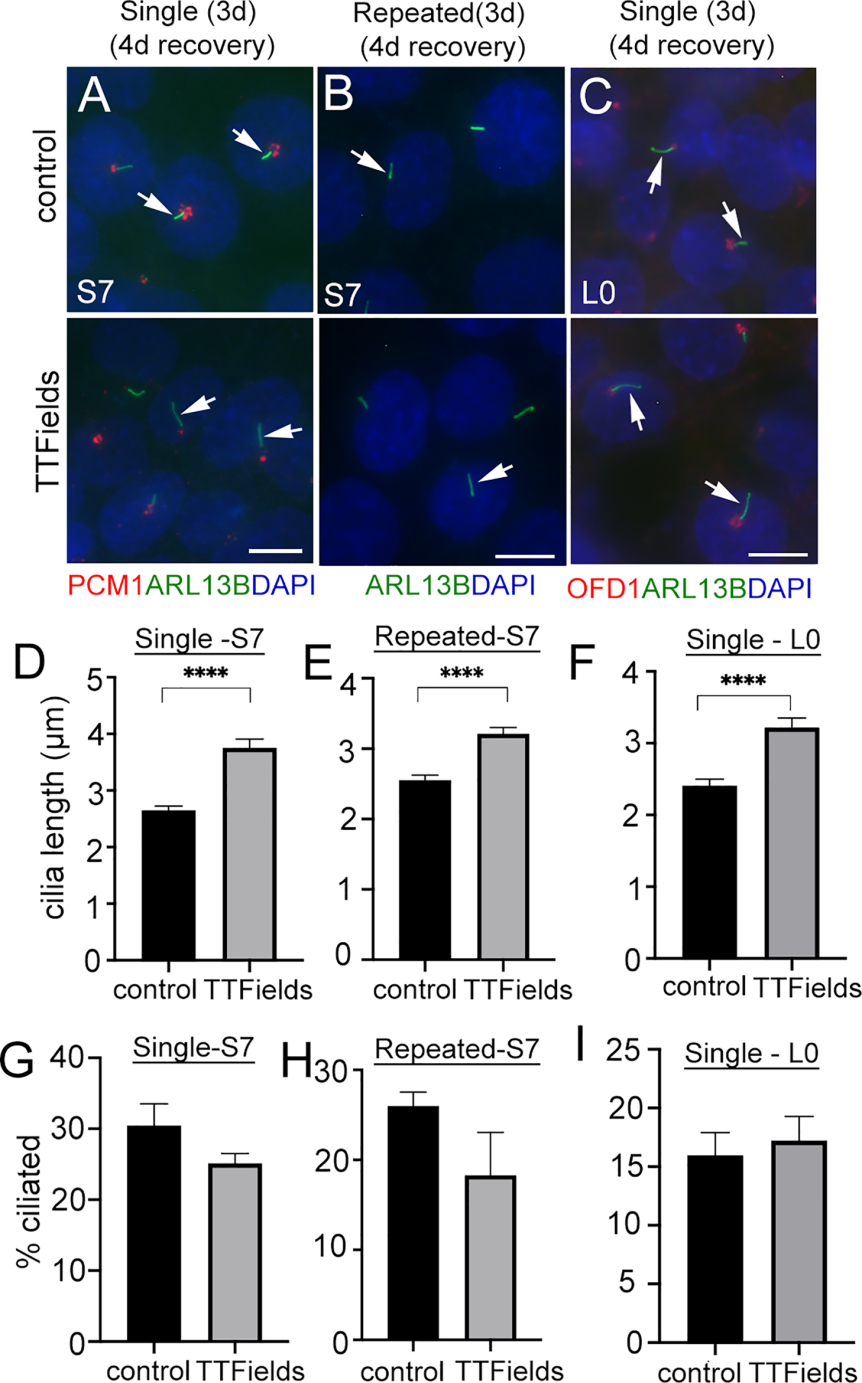
Similar frequencies of elongated glioma cilia after halting TTFields treatment. **(A, B)** S7 and **(C)** L0 cells without (control) or with a single or repeated exposure to 3 days (3d) of TTFields treatment then dissociated onto coverslips for 4 days. Fixed cells were immunostained for ARL13B (green), PCM1, or OFD1 (red) with nuclei labeled with DAPI (blue). Control and TTFields-treated cells showed ARL13B^+^ cilia (arrows) extending from the PCM1^+^ or OFD1^+^ puncta which concentrates around basal bodies. Scale bar = 10 µm. Mean lengths (µm) of **(D, E)** S7 and **(F)** L0 ARL13B^+^ cilia after 4 days recovery from a single or repeated exposure to TTFields. Percent of ciliated cells in **(G, H)** S7 and **(I)** L0 after 4 days recovery from a single or repeated exposure to TTFields. ^****^ *p* < 0.0001 (ANOVA).

The significant depletion of primary cilia by TTFields led us to ask if this effect was permanent. That is, would the frequency of ciliated glioma cells remain low if treatment is stopped and cells are allowed to recover? After single or repeated TTFields exposure, we plated cells in serum for 4 days, fixed, and immunostained for ARL13B alone or combined with pericentriolar material 1 (PCM1), another protein that clusters around the basal body and centrioles in glioma cells (29), or OFD1. In S7 cells, ARL13B+ cilia were detectable but appeared longer than control (**Figures 2A, B** **)**. Quantification of lengths of ARL13B^+^ cilia demonstrated a significant increase in the TTFields-exposed groups (**Figures 2D, E** **)**. A similar increase in length in L0 was observed after recovery from TTFields (**Figures 2C, F** **)**. However, in either S7 or L0 cells, there was no change in cilia frequency after recovery from TTFields (**Figures 2G–I**). These data indicate that frequencies of ciliated glioma cells are restored after TTFields but are affected in a way that leads to elongation.

### TTFields Do Not Have the Same Impact on Normal Mouse Neural Cilia

Considering the robust depletion of glioma cilia within 24 h of exposure to TTFields, we next asked whether cilia of normal primary neural cell types are similarly affected by TTFields. To test this, we cultured dissociated mouse embryonic cortices on glass coverslips for 11 days *in vitro* (DIV). At 11DIV, we assigned coverslips as control or TTFields (24 h or 3 days exposure). One advantage of this type of culture is that we can examine the effects of TTFields on cells that differentiate into various subtypes including astrocytes and neurons, marked by glial fibrillary acidic protein (GFAP) and neuronal nuclei (NeuN) expression, respectively.

**Figure 3:**
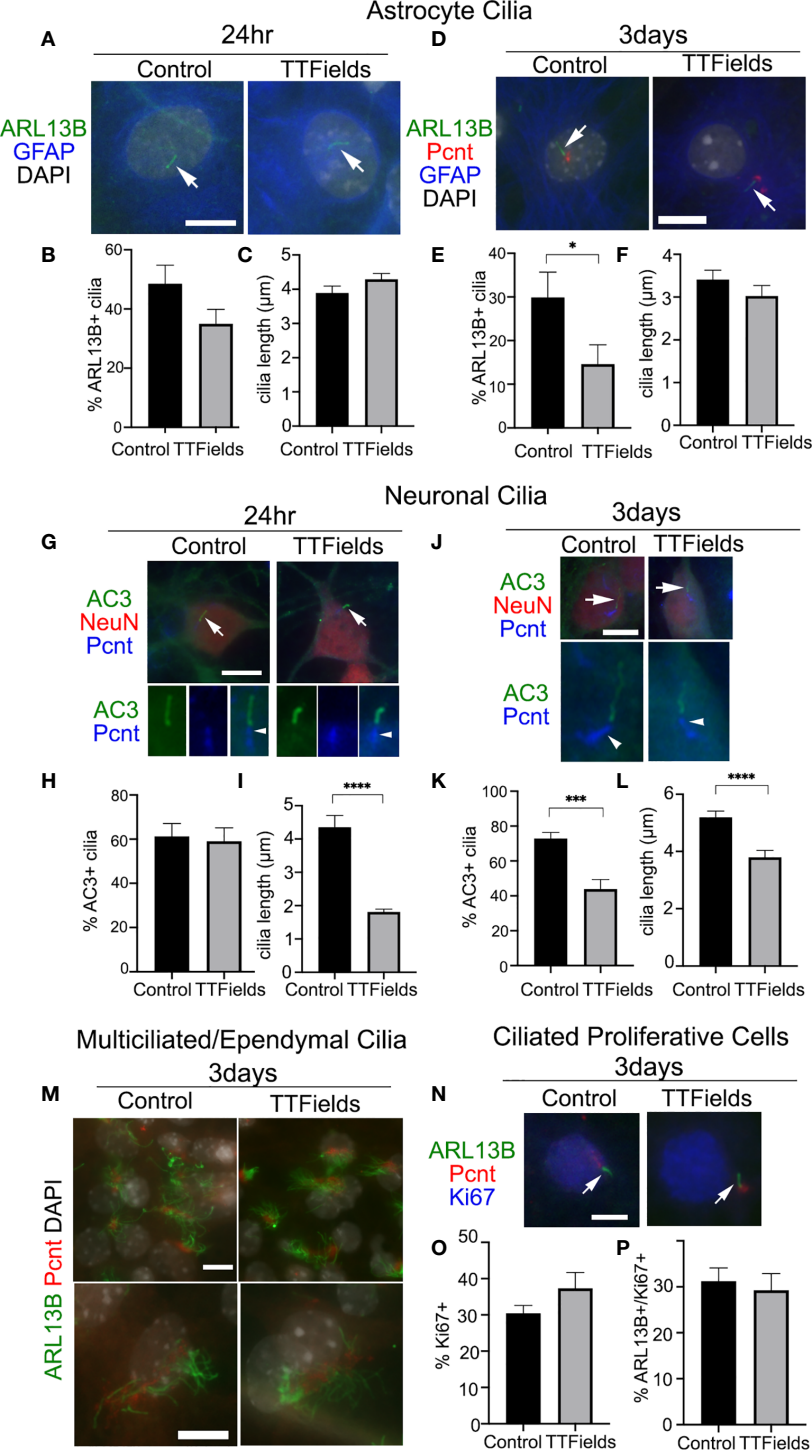
Effects of TTFields on normal mouse neural cell types *in vitro*. Mixed primary cultures from neonatal mouse cerebral cortex were dissociated and maintained for 11DIV and left untreated (Control) or exposed to 24 h or 3 days of continuous TTFields and fixed. **(A)** Cells were stained for ARL13B (green) and GFAP (blue) after 24 h TTFields. Nuclei were labeled with DAPI. Arrows point to ARL13B^+^ cilia in both groups at 24 h. **(B)** Percent of GFAP^+^ cells with ARL13B^+^ cilia and **(C)** mean length of ARL13B^+^ cilia on GFAP^+^ cells after 24 h. **(D)** Cells were stained for ARL13B (green), pericentrin (Pcnt, red), and GFAP (blue) after 3 days of TTFields. **(E)** Percent of GFAP^+^ cells with ARL13B^+^ cilia and **(F)** mean length of ARL13B^+^ cilia on GFAP^+^ cells after 3 days. **(G)** Cells were stained for type 3 adenylyl cyclase (AC3) (green), NeuN (red), and Pcnt (blue) after 24 h TTFields. Nuclei were labeled with DAPI. The arrows in the upper panels point to cilia enlarged below each image showing Pcnt^+^ basal bodies for indicated cilia. **(H)** Percent of NeuN^+^ cells with AC3^+^ cilia and **(I)** mean length of ARL13B^+^ cilia on NeuN^+^ cells after 24 h TTFields. **(J)** Cells were stained for AC3 (green), NeuN (red), and Pcnt (blue) after 3 days of TTFields. The arrows in the upper panels point to cilia enlarged below each image which shows the Pcnt^+^ basal bodies for indicated cilia. **(K)** Percent of NeuN^+^ cells with AC3^+^ cilia and **(L)** mean length of ARL13B^+^ cilia on NeuN+ cells after 3 days of TTFields. **(M)** Cells were stained with ARL13B (green) and Pcnt (red) with nuclei labeled with DAPI (white). Lower magnification (upper panels) and enlarged (lower panels) examples of multiciliated cells in control (left panels) and 3 days of TTFields (right panels). Bars = 10 µm. **(N)** Cells were stained for ARL13B (green), Pcnt (red), and Ki67 (blue) after 3 days of TTFields. Images show examples of Ki67^+^ nuclei with ARL13B^+^ cilia (arrows) extending from Pcnt^+^ basal bodies. **(O)** Percent of Ki67^+^ cells per field analyzed in each group. **(P)** Percent of Ki67^+^ cells with ARL13B^+^ cilia. ^*^ *p* < 0.05; ^***^ *p* < 0.001; ^****^ *p* < 0.0001 (ANOVA).

We first examined astrocyte cilia through a combined immunostaining for GFAP, ARL13B, and pericentrin (Pcnt, a protein concentrated around the cilia basal body) (**Figures 3A, D** **)**. After 24 h of TTFields, we did not observe significant differences in the frequency (**Figure 3B**) or length (**Figure 3C**) of astrocyte cilia. However, after 3 days of TTFields, there were significantly fewer ciliated GFAP^+^ cells (**Figure 3E**), though the lengths of these cilia were comparable with control (**Figure 3F**). These data suggest that, at least at acute timepoints after TTFields, astrocyte cilia appear more resistant to TTFields than glioma cells.

Next, we examined neuronal cilia by triple immunostaining for NeuN, type 3 adenylyl cyclase (AC3), an enzyme enriched in most neuronal cilia in the cortex (44–46), and Pcnt (47, 48) (**Figures 3G, J** **)**. After 24 h of TTFields, we did not observe any significant changes on the frequency of neurons with AC3^+^ cilia (**Figure 3H**), but the lengths of AC3^+^ cilia were significantly reduced (**Figure 3I**). After 3 days of TTFields, both the frequency and length of AC3^+^ cilia on NeuN+ cells were significantly reduced compared with control (**Figures 3K, L**, respectively). The extent of the reduced ciliary frequency was ~40% of control neurons, compared with about a 90% decrease in L0 cells after similar duration. Thus, neurons, especially after 24 h of TTFields, appear more resistant though not completely spared from the effects of TTFields.

The neural cultures also contained populations of multiciliated cell types (presumably cells that differentiated into ependymal cells) and proliferating cells. However, the cells bearing tufts of cilia, detected by a combination of ARL13B and Pcnt, appeared comparable after 24 h (data not shown) and 3 days of continuous TTFields (**Figure 3M**). A fraction of the cells in the culture were still Ki67^+^, suggesting they were still active in the cell cycle (**Figure 3N**). However, among the Ki67^+^ cells (**Figure 3O**), we did not observe a significant change in the percentage of ciliated cells (**Figure 3P**). Thus, TTFields seems to spare the ability for multiciliated cells and ciliated cycling cells to form or maintain their cilia. Overall, these data suggest a differential sensitivity to TTFields between normal neural cell types and glioma cells.

### TTFields Induction of Autophagy and Death of Ciliated Cells Contribute to Cilia Depletion

**Figure 4:**
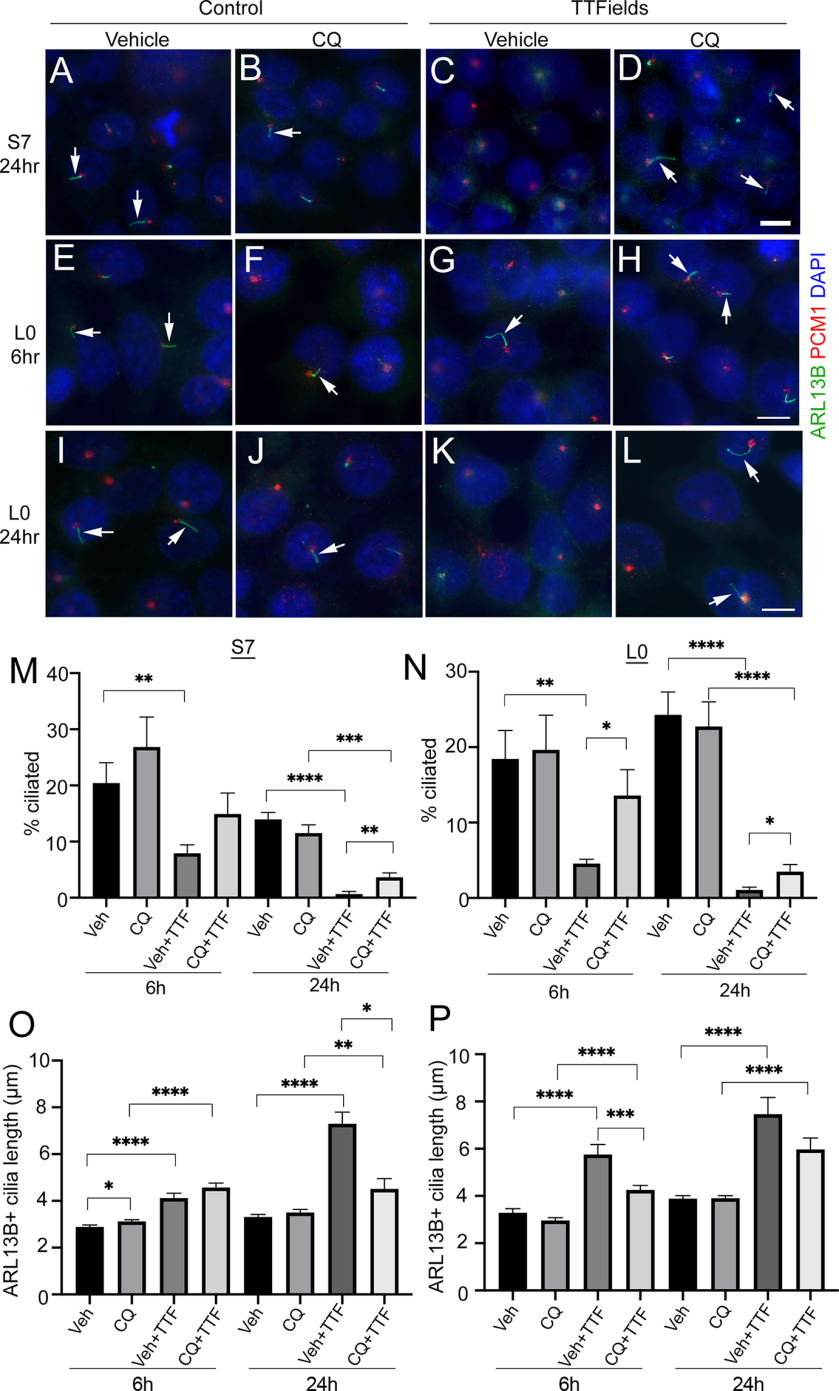
Chloroquine pretreatment partially prevents TTFields-induced loss of cilia. **(A–D)** S7 or **(E–L)** L0 cells pretreated with vehicle **(A, C, E, G, I, K)** or 20 µM chloroquine (CQ) **(B, F, J, D, H, L)** and left untreated (control) **(A, B, E, F, I, J)** or exposed to TTFields **(C,D, G, H, K, L)**. Cells were fixed after the indicated time point and stained for ARL13B (green) and PCM1 (red) with nuclei stained with DAPI (blue). Arrows point to cilia observed in the indicated treatments. Scale bars for the respective rows in **(D, H, L)** = 10 µm. **(M)** Percent of S7 and **(N)** L0 cells with ARL13B^+^ cilia for the indicated treatment group after 6 h or 24 h of TTFields (TTF). Mean lengths of ARL13B^+^ cilia in **(O)** S7 or **(P)** L0 cells for the indicated treatment group after 6 or 24 h of TTFields (TTF). ^*^ *p* < 0.05; ^**^ *p* < 0.01; ^***^ *p* < 0.005; ^****^ *p* < 0.0001 (ANOVA).

What is the mechanism through which TTFields promotes rapid cilia loss in glioma cells? A number of factors and pathways promote cilia disassembly (49). Examples include calcium shock/influx (50), or autophagy activation (51), processes that have been shown to rapidly increase after TTFields onset (14, 17). We examined whether buffering extra/intracellular Ca^2+^ by pretreating cells with 600 mM EGTA or 1 µM BAPTA increases cilia frequency during TTFields; however, we did not observe any prevention of cilia loss (data not shown). We then examined if the autophagy pathway changes at cilia were involved, in part because the reduced OFD1 expression we observed around the basal bodies/centrioles after TTFields (**Figure S1**), is a potential indicator of autophagy activation (42, 52, 53). OFD1 is also an autophagy receptor that controls the early phases of the autophagy cascade and autophagosome biogenesis (54). In addition, we observed microtubule-associated proteins 1A/1B light chain 3B (LC3B) and phospho-AMPK (pAMPK) recruitment to basal bodies after single and repeated TTFields application (**Figure S4**) consistent with reports that autophagy proteins are recruited to primary cilia (51, 55). Thus, we pretreated S7 and L0 cells 30 min before TTFields induction with vehicle or the autophagy inhibitor chloroquine (CQ) (20 µM), fixed cells after 6 or 24 h, and analyzed the frequency and length of cilia. A concentration of 20 µM CQ was selected because it inhibited autophagy pathway activation in response to TTFields in U87 and other glioma cell lines (14). We found that S7 glioma cells pretreated with CQ 30 min before TTFields led to a significant increase in the percent of ciliated cells after 24 h compared with control (**Figures 4A–D, M** **)**. The TTFields-induced increase in cilia length in S7 cells was also reduced by CQ at 24 h (**Figure 4O**). In L0 cells, we observed significantly more ciliated cells after 6 h (**Figures 4E–H, N** **)** and 24 h (**Figures 4I–L, N** **)** pretreatment with CQ and exposed to TTFields. In addition, cilia length of CQ-treated L0 cells at 6 h was significantly reduced compared with vehicle after 6 h TTFields (**Figure 4P**), suggesting autophagy activation may be underlying the observed elongation after TTFields. It is noteworthy that in both S7 and L0 cells, 6 h of TTFields was sufficient to observe significant cilia elongation. Furthermore, because CQ did not fully restore the frequency of cilia to control suggests that either CQ may not inhibit autophagy in all cells, or that the activation of autophagy may represent one of the factors resulting in TTFields-induced cilia depletion.

To more directly examine how ciliated cells respond to TTFields, we transfected L0 and S7 cells with two cDNA constructs encoding ARL13B:GFP and OFD1:mCherry allowing us to track isolated cells displaying ARL13B:GFP^+^ cilia with OFD1:mCherry^+^ clusters around the basal body overnight (**Figure S5A**). We live-imaged cells up to 24 h after transfection using Novocure’s Inovitro LIVE imaging system. Notably within several hours after TTFields onset, we observed ciliated L0 cells that appeared to die (**Figure S5B, C**), with similar observations in S7 cells during TTFields (**Figure S5D**). These data indicate TTFields may have a direct impact on the survival and proliferation of ciliated glioma cells or cells that are derived from ciliated glioma cells.

### TMZ-Induced Ciliogenesis Is Blunted by TTFields

The survival benefit promoted by TTFields in patients occurs during TMZ maintenance therapy (3, 4). TMZ has recently been reported to increase the frequency and length of ARL13B+ cilia patient-derived glioblastoma cells (30). Thus, we asked if the effects of TTFields on cilia would be similar in the presence of TMZ chemotherapy. The effects of TMZ on glioma ciliogenesis have not been extensively analyzed with respect to different cell lines and different concentrations and durations of exposure. Thus, we first examined how different durations and concentrations of TMZ, doses lower than those typically used to kill cells in *in vitro* assays, affect the frequency and length of ARL13B+ glioma cilia in our cell lines.

**Figure 5:**
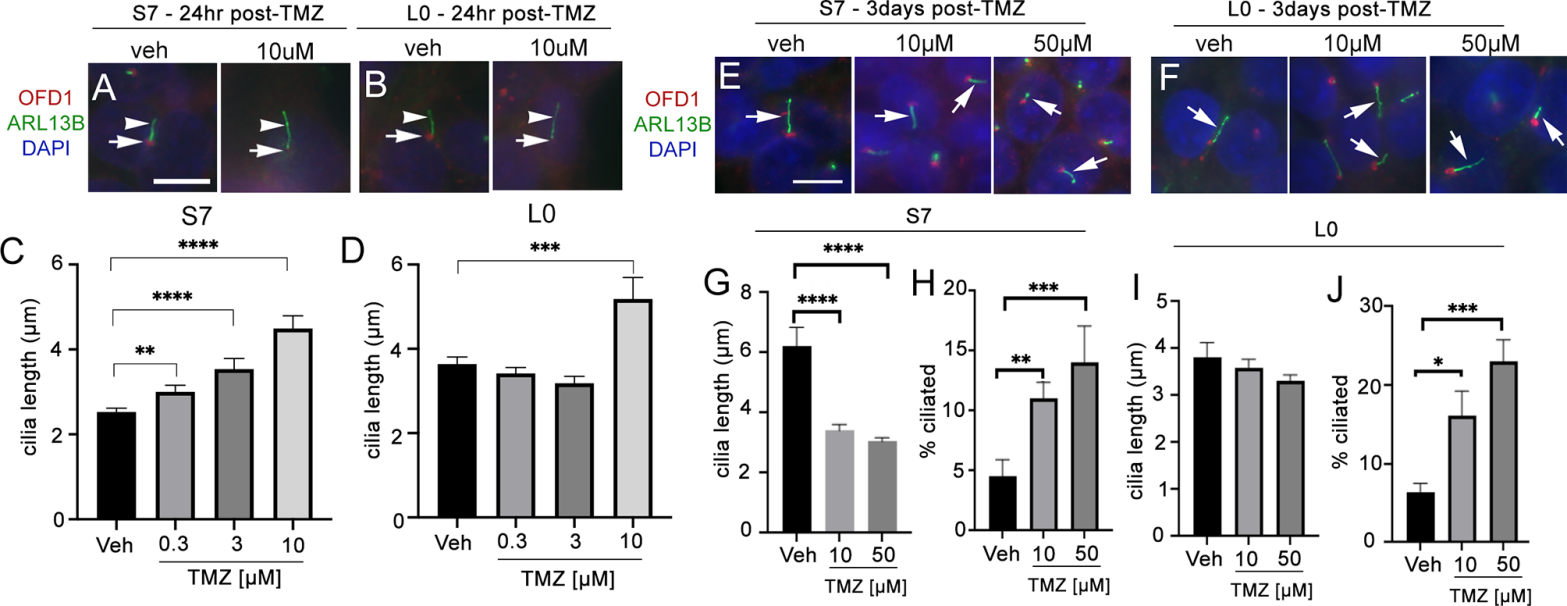
Temozolomide stimulates ciliogenesis in L0 and S7 cells. **(A)** S7 and **(B)** L0 cells were grown for 24 h in vehicle or 10 µM TMZ. Cells were fixed and immunostained for OFD1 (red) and ARL13B (green). Nuclei were labeled with DAPI. TMZ-treated cells appeared to show elongated ARL13B^+^ cilia (arrowheads) and OFD1 intensity at the base of the cilia (arrows) appeared to decrease compared with vehicle in both cell lines. Scale bar = 10 µm. Mean lengths of cilia in **(C)** S7 and **(D)** L0 cells after a 24-h exposure to the indicated concentration (µM) of TMZ. **(E)** S7 and **(F)** L0 cells were grown for 72 h after one treatment of vehicle, 10 or 50 µM TMZ. Cells were fixed and immunostained for OFD1 (red) and ARL13B (green). Arrows point to ARL13B^+^ cilia in each treatment. Mean lengths of ARL13B^+^ cilia in **(G)** S7 or **(I)** L0 cells 3 days after exposure to the indicated concentration (µM) of TMZ. Percent of ARL13B^+^ cilia in **(H)** S7 or **(J)** L0 cells 3 days after exposure to the indicated concentration (µM) of TMZ. *p < 0.05, **p < 0.01, ***p < 0.005, ****p < 0.0001 (ANOVA).

In L0 and S7 cells, 10 µM TMZ appeared to elongate primary cilia in both cell lines 24 h after treatment (**Figures 5A, B** **)**. In S7 cells, we observed a dose-dependent effect with 0.3, 3, and 10 µM TMZ sufficient to increase cilia length 24 h after exposure (**Figure 5C**). In L0 cells, we found that 10 µM TMZ significantly increased the length of ARL13B^+^ cilia compared with lower TMZ concentrations and vehicle (**Figure 5D**). Three days after exposure to TMZ (a duration chosen because we performed 3 days of continuous TTFields), we found that in S7 cells, 10 and 50 µM TMZ significantly reduced the length of cilia (**Figures 5E, G** **)** but significantly increased the frequency of ciliated cells (**Figure 5H**). However, 3 days after TMZ exposure in L0 cells did not affect cilia length after 10 or 50 µM (**Figures 5F, I** **)** but significantly increased the frequency of ciliated L0 cells (**Figure 5J**) as in S7 cells. These results support results of recent studies (30) and suggest TMZ is capable of stimulating the elongation of ARL13B^+^ cilia, at least acutely, and increasing the frequency of ciliated glioma cells.

**Figure 6:**
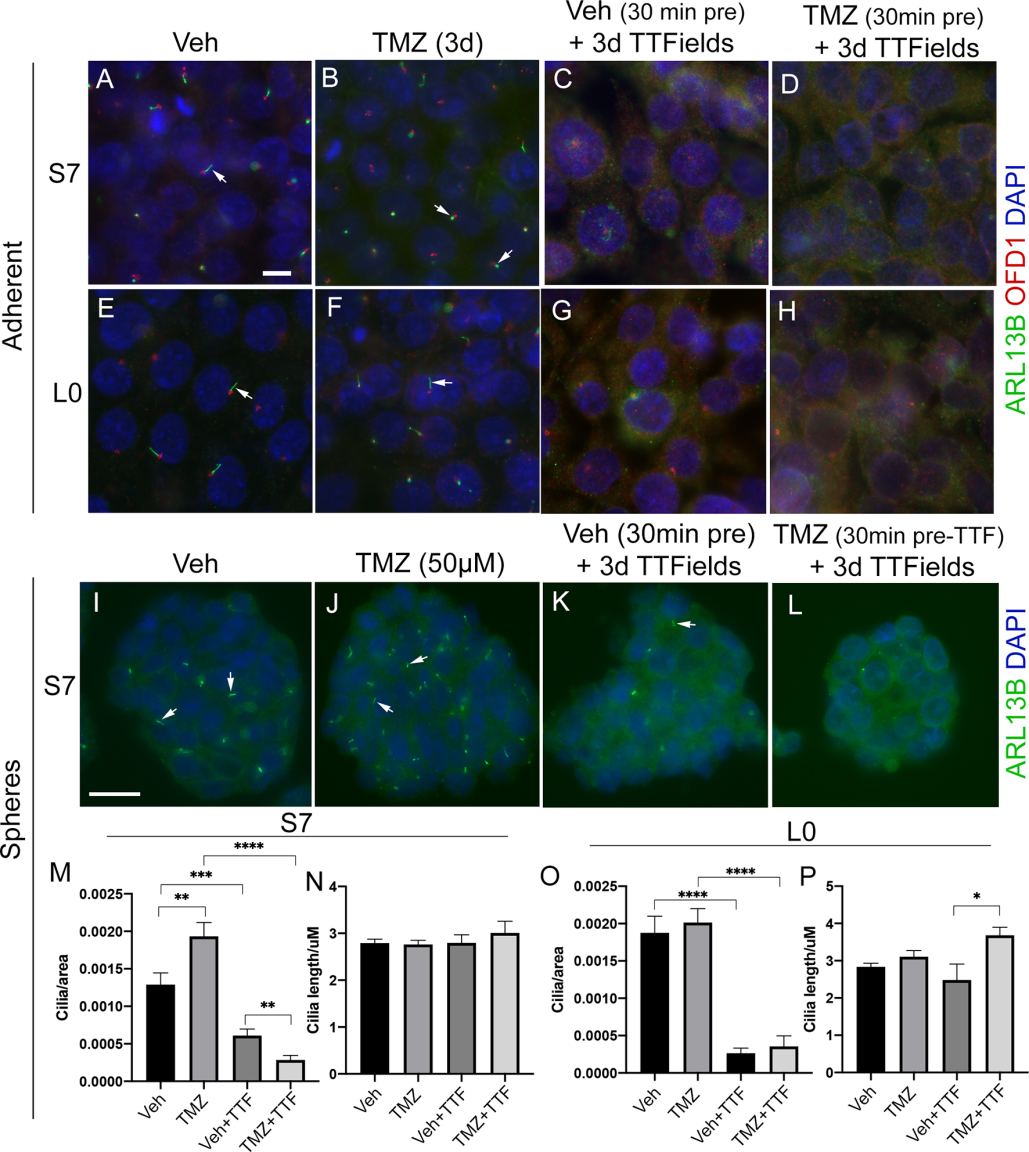
TTFields blocks the TMZ-mediated increase in ciliogenesis. Adherent S7 cells pretreated with **(A, C)** vehicle or **(B, D)** 10 µM TMZ and left untreated (control) **(A, B)** or exposed to 3 days of TTFields **(C, D)**. Adherent L0 cells pretreated with **(E, G)** vehicle or **(F, H)** 20 µM TMZ and left untreated (control) **(E, F)** or exposed to 3 days of TTFields **(G, H)**. S7 and L0 cells were fixed and stained for ARL13B (green) and OFD1 (red). Nuclei are labeled with DAPI (blue). Spheres of S7 cells pretreated with **(I, K)** vehicle or **(J, L)** 50 µM TMZ and left untreated (control) **(I, J)** or exposed to 3 days of TTFields **(K, L)**. To examine cilia, spheres were fixed, sectioned, and stained for ARL13B (green, arrows). The number of cilia/per area (µm^2^) of traced sections of **(M)** S7 or **(O)** L0 spheres for the indicated treatment. The mean length (µm) of cilia in **(N)** S7 or **(P)** L0 spheres for the indicated treatment. ^*^ *p* < 0.05; ^**^ *p* < 0.01; ^***^ *p* < 0.001; ^****^ *p* < 0.0001 (ANOVA). Scale bars (in µm) in **(A)** = 10 and **(I)** = 20.

Since TMZ generally stimulates ciliogenesis, and TTFields inhibit it, we examined glioma cilia with a combination of these treatments in adherent cells and spheres (**Figure 6**). First, we pretreated adherent S7 and L0 cells with concentrations of TMZ that stimulated ciliogenesis about 30 min before a 3-day exposure to TTFields. We found that in both adherent S7 (**Figures 6A** **–D**) and L0 (**Figures 6E** **–H**) cells, ciliogenesis was not observed in the presence of TMZ plus TTFields (**Figures 6D, H** **)**. We also examined if TTFields had the same effect in gliomaspheres. We cultured S7 and L0 spheres for 3 days, and then treated them for 3 days with vehicle, 50 µM TMZ, TTFields, or TTFields plus 50 µM TMZ (added 30 min before onset) (**Figures 6I** **–L**). We then collected and fixed spheres, and sectioned and immunostained them for ARL13B. For each sphere, we normalized the number of cilia to the area of the sphere. In S7 cells, we found that TMZ alone increased the frequency of cilia (**Figure 6M**), consistent with what we observed in adherent cells (**Figure 5E**). As expected, TTFields significantly reduced the frequency of cilia in the spheres but TMZ-induced increase did not occur in the presence of TTFields (**Figure 6M**). Interestingly, we did not observe a change in the length of cilia across groups (**Figure 6N**). Unlike S7 cells, TMZ alone did not increase the frequency of cilia in L0 spheres (**Figure 6O**), and TMZ plus TTFields cotreatment increased cilia lengths compared to vehicle plus TTFields (**Figure 6P**). However, like S7 cells, TTFields reduced the frequency of cilia in the L0 spheres which was not enhanced by the cotreatment of TMZ plus TTFields (**Figure 6O**). Together, these results indicate that TTFields disrupt the prociliogenic effects of TMZ.

### The Combined Efficacy of TMZ and TTFields Correlates With the Timing of Treatment and ARL13B^+^ Cilia

**Figure 7:**
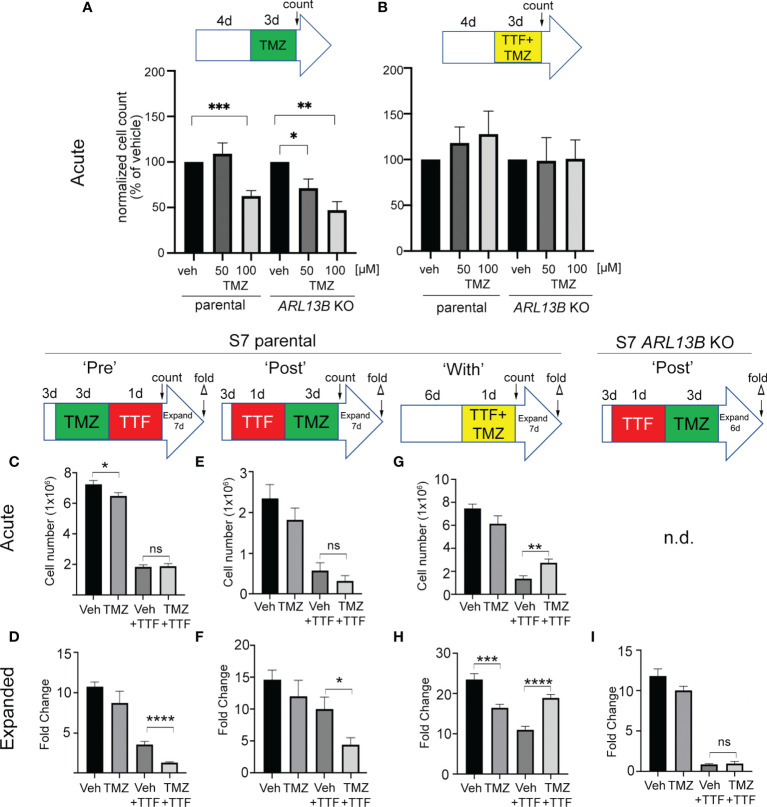
Combined TMZ and TTFields efficacy correlates with treatment sequence and ARL13B^+^ cilia. **(A, B)** S7 parental or *ARL13B* KO (clone G12) cells were grown as spheres for 4 days and then treated with vehicle (veh) or 50 or 100 µM TMZ and exposed to an additional 3 days without **(A)** or with TTFields. Bar graphs show the normalized cell number (% of control) at the end of 7 days. **(C, D)** TMZ “pre”-experiment: S7 parental cells were treated with 100 µM TMZ for 3 days before a 24-h treatment of TTFields or control (con). Cells were counted immediately after this treatment (acute count), and then pooled, and 2.5 × 10^4^ cells/well were expanded in fresh media for 7 days in 24-well plates (*n* = 12 wells/group), and the fold expansion was calculated (fold count) **(D)**. **(E, F)** TMZ “post”-experiment: TMZ (100 µM) was given for 3 days after a 24-h TTFields treatment with S7 cells being counted acutely **(E)** or expanded in fresh media for 7 days **(F)**. **(G, H)** TMZ “with” experiment: S7 cells were grown for 6 days and then simultaneously treated with TMZ (100 µM) and TTFields for 24 h, counted acutely **(G)** or examined for fold expansion after 7 days **(H)**. **(I)** Similar experiment as **(F)** but examining fold expansion of S7 *ARL13B* KO cells after 6 days. n.d., not determined; ns, not significant; ^*^ *p* < 0.05; ^**^ *p* < 0.01; ^***^ *p* < 0.001; ^****^ *p* < 0.0001 (ANOVA).

Previously, we found that deleting key ciliogenesis genes (e.g., *PCM1*, *KIF3A*) enhanced sensitivity of glioma cells to TMZ (29). Similarly, glioma cells expressing ARL13B shRNA, which depleted cilia, were more sensitized to TMZ *in vitro* and *in vivo* (30). Using our S7 glioma transgenic cell line depleted in ARL13B and cilia using CRISPR/Cas9 (32), we examined how these cells proliferated in response to TMZ, TTFields, or TMZ and TTFields cotreatment. After 4 days of growing S7 parental or ARL13B KO spheres, we treated them with vehicle or 50 or 100 µM TMZ in the absence or with 3 days of TTFields (**Figures 7A** **, B**). In parental S7 cells, proliferation was reduced in 100 µM TMZ group (**Figure 7A**). However, in *ARL13B* KO cells, proliferation was significantly reduced at 50 and 100 µM TMZ groups (**Figure 7A**), consistent with results of previous studies that ARL13B^+^ cilia are associated with resistance to TMZ. However, when we cotreated S7 parental or *ARL13B* KO cells with TMZ and TTFields, there was no added toxicity in either group (**Figure 7B**). This suggests that in cells with cilia ablation *via* treatment with TTFields or thru genetic means, the cotreatment of TMZ and TTFields may not lead to an acute additive toxicity.

Surprised that 3 days of TMZ and TTFields cotreatment in S7 parental cells did not show additive toxicity and trended towards increased proliferation (**Figure 7B**), we wondered if potential cotoxicity relates to treatment sequence or the treatment effect could be delayed. For example, would stimulating ciliogenesis with TMZ render more cells susceptible to TTFields, and/or would TTFields suppression of ciliogenesis sensitize more glioma cells to TMZ? To test this, we administered TMZ before (PRE) (**Figures 7C, D** **)**, after (POST) (**Figures 7E, F** **)**, or during (WITH) (**Figures 7G, H** **)** a 24-h window of TTFields application. In the PRE-experiment, we found that the acute numbers of TMZ + TTFields-treated cells were similar to vehicle (Veh) + TTFields (**Figure 7C**). However, the fold expansion of TMZ + TTFields-treated cells 7 days after treatment was significantly reduced compared with Veh + TTFields (**Figure 7D**). In the POST-experiment, we also did not observe an acute reduction of TTFields + TMZ-treated cells compared with control (**Figure 7E**) but did observe a significant reduction of TTFields-TMZ-treated cells 7 days after treatment (**Figure 7F**). Surprisingly, in the “WITH” experiment, we observed an acute increase in TMZ + TTFields-treated cells compared with Veh + TTFields (**Figure 7G**), and a significant increase in the expansion of TMZ + TTFields cotreated cells 7 days after treatment (**Figure 7H**). Thus, TMZ given pre- or post-TTFields slows tumor cell recurrence, but coadministration enhanced tumor cell recurrence. We next asked if either of the treatment paradigms that slowed tumor cell recurrence correlated with the presence of ARL13B^+^ cilia. Since we observed a significant effect of giving TMZ after TTFields on parental cells, we examined fold expansions of S7 *ARL13b* KO cells 6 days after TTFields + TMZ treatment (**Figure 7I**). However, there was no significant difference in the expansion between TTFields + TMZ-treated and TTFields + Veh-treated ARL13B KO cells (**Figure 7I**). These results suggest that the relative timing of TMZ exposure to TTFields impacts subsequent tumor cell expansion *in vitro* that also correlates with the presence of ARL13B^+^ cilia.

### TTFields Disrupt Primary Cilia in Patient Tumors *Ex Vivo*

**Figure 8:**
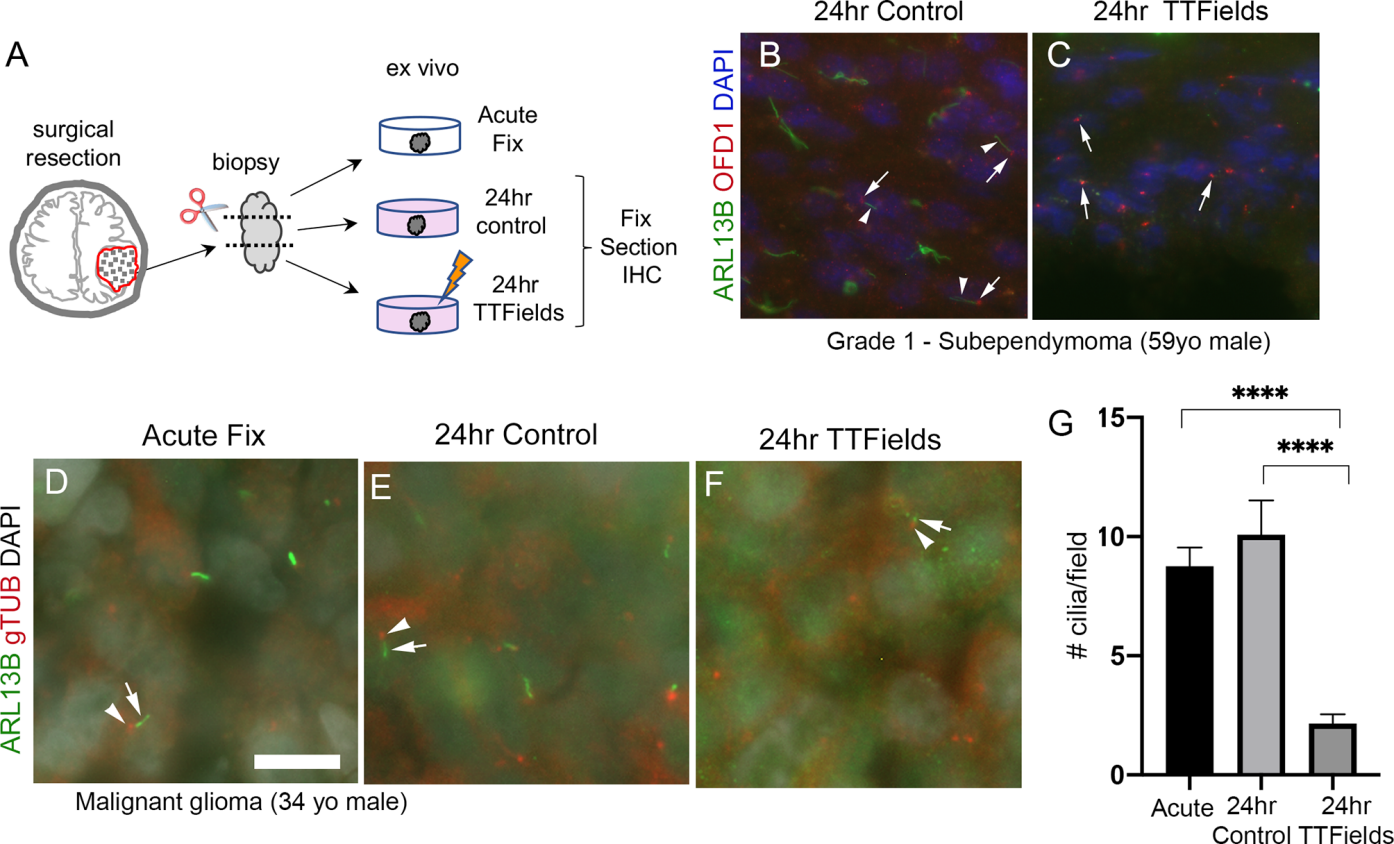
TTFields disrupt cilia in patient samples *ex vivo*. **(A)** *Ex vivo* treatment of surgical resections. Biopsies were dissected and separated into immediate/acute fix, 24 h control or 24 TTFields treatment. Tissue was fixed, frozen, cryosectioned, and immunostained. Nuclei are labeled with DAPI. **(B)** Immunostaining of control section of a grade 1 subependymoma showed ARL13B^+^ cilia (arrowheads) extending from OFD1^+^ basal bodies (arrows). **(C)** TTFields-treated tissue section showed OFD1^+^ basal bodies (arrows) without obvious ARL13B^+^ cilia extensions. **(D–F)** Tissue from a malignant glioma from a 34-year-old male that was separated into immediate/acute fixation, 24-h control, or 24-h TTFields exposure. Tissues were fixed, cryosectioned, and immunostained for ARL13B (green) and gTub (red), and nuclei labeled with DAPI (white). ARL13B^+^ cilia (arrows) with gTUB^+^ basal bodies (arrowheads) are readily detected in acute **(D)** and 24-h control **(E)** but appeared blunted or generally absent in TTFields group **(F)**. **(G)** Mean (+/− SEM) number of cilia/field (*n* = 12–13 fields/group) from samples in **(D–F)**. ^****^ *p* < 0.0001 (ANOVA). Scale bars (in µm) in **(A)** = 10.

While we currently do not have the technical capability to test TTFields in an intracranial, xenograft model, we asked if the effects of TTFields on cilia we observed in adherent or spheres of glioma cells are detectable within the patient tumor microenvironment. To test this, we divided fresh biopsy samples into 3 groups: immediate/acute fixation, 24-h control, or 24 h of TTFields. We then fixed and immunostained cryosections of biopsies (**Figure 8A**). First, we examined a subependymoma (a grade 1 glioma), a tumor type reported to possess cilia (56). We found that ARL13B^+^ cilia extending from OFD1^+^ basal bodies were readily detectable in control (**Figure 8B**) whereas we only clearly observed OFD1^+^ basal bodies in the TTFields-treated biopsy (**Figure 8C**). We also received newly diagnosed GBM biopsies from a 34-year-old man and a 66-year-old man which we treated similarly except for the immunostained basal bodies/centrioles with gTUB and cilia with ARL13B antibodies. We observed gTUB^+^ basal bodies with ARL13B^+^ cilia in acutely fixed tissue and in overnight control (**Figures 8D, E, G** **)**, whereas the cilia appeared blunted or generally reduced in TTFields-treated tissue (**Figures 8F, G** **)**. Similar observations were made in a GBM biopsy of a 66-year-old man exposed to 24 h of TTFields (**Figure S6**). These data are consistent with our cultured adherent cells and gliomaspheres, raising the possibility that TTFields disrupts ARL13B^+^-ciliated tumor cells within the tumor.

## Discussion

We show that TTFields significantly impact the ability of glioma cells to maintain their primary cilium. Low- and high-grade glioma cells disassemble their cilia shortly after TTFields, though the population is not completely eliminated because ciliated cells reappear at similar frequencies if the treatment is stopped. The mechanism leading to cilia loss appears to involve a combination of autophagy activation and death of ciliated cells. TTFields does not similarly impact the cilia of various normal neural cell types, pointing to one aspect of glioma biology that may be differentially sensitive to TTFields. Furthermore, TMZ-induced increase in ciliated glioma cells is inhibited by TTFields. This is of potential significance because ARL13B-mediated signaling associated with glioblastoma cilia is linked to both tumor growth and TMZ resistance *in vivo* (30). Thus, disruption of cilia by TTFields may help enhance TMZ efficacy or help eliminate a population of treatment-resistant cells. Surprisingly, we found that simultaneous TMZ and TTFields treatment is less effective on slowing tumor cell recurrence than TMZ added before or after TTFields, an effect that is lost on cilia-depleted tumor cells. Considering our observation that TTFields-mediated changes in cilia within patient tumors raise the possibility that tumors containing high levels of ciliogenesis could be more receptive to TTFields and TMZ, future studies are needed to correlate tumor ciliation, TTFields, and TMZ on patient outcome.

How does TTFields lead to glioma primary cilia dismantling? Within hours, TTFields triggers axonemal elongation with accompanying changes in the distribution of proteins along the ciliary membrane, culminating in the loss of the cilia. The redistribution of ciliary membrane-associated proteins may be due to the effect of TTFields on membrane permeability reported to occur during the same timescale (16). It is not clear if TTFields cause cilia to be absorbed back into the cell, shed into the extracellular milieu, or both. Most mammalian cilia appear to disassemble by shedding the whole cilium (57, 58). However, live imaging studies of glioma cells during TTFields support a withdrawal or absorption back into the cell as we did not observe cilia detachment. Whatever the mechanism of cilia loss after TTFields, the changes in cilia may serve as a biomarker of the efficacy of TTFields in the tumor. Although TTFields eliminates cilia/ciliated glioma cells, they grow back at the same frequency though longer if treatment is stopped. We do not know if the regrown cilia are from the same cells or represent a new population of ciliated cells that is more sensitive or resistant to treatment, which could potentially be addressed *via* extended live imaging during and after TTFields. It is important to note that cilia are dynamic structures and generally assembled during the G0/G1 phase of the cell cycle, and then disassembled prior to and during M-phase (49, 59). Thus, ciliated cells we observed after TTFields may be from cells still going through or coming out of M-phase after TTFields. Indeed, a majority of isolated GBM cell clones (~60%–90%) can form ciliated populations (38). Future studies could address this issue in a more detailed manner using an approach that monitors cell cycle and cilia simultaneously (e.g (59).,) in live cells before, during, and after TTFields. The ciliary lengthening on recovered cells could be due to elevated autophagy pathway activation, which has been shown to elongate cilia (42). CQ has been observed to reduce the autophagy-mediated increase in cilia length on human kidney proximal tubular cells (60), which is further supported by our observation that CQ reduces the TTFields-induced ciliary lengthening (**Figures 4O, P** **)**.

Autophagy activation may represent one contributing factor in the disappearance of cilia after TTFields, since CQ pretreatment did not fully restore the frequency of ciliated cells. TTFields may trigger many other factors that promote cilia removal. It is possible that only a fraction of the cilia is removed by autophagy. HDAC6-mediated autophagy can result in “ciliophagy” in mouse tracheal epithelial cells and cholangiocarcinoma cells (51, 61), and thus some glioma cilia may be driven by HDAC6-mediated autophagy whose signaling at cilia is a key regulator of glioma cell proliferation (32). Whatever the mechanism of autophagy linked to glioma cilia, it is unclear whether it is promoting or reducing cell survival. However, the link to autophagy is noteworthy as TTFields activation of autophagy appears to have dual significance. On one hand, it may drive the death of cells (11) or alternatively promote activation of pathways that reduce sensitivity to TTFields (14). A scenario in which the ciliated glioma cells die by TTFields favors the former, whereas if glioma cells regrow, their cilia (supporting the return of cilia frequency) favor the latter. Alternatively, there could be a mixture of these scenarios that requires further study.

It is not clear why normal differentiating or proliferating neural cilia are less affected by TTFields. It is possible that plasma membrane of normal mouse neural cell type is more impervious to or recovers quicker from the membrane-permeating effects of TTFields than glioma ciliary membranes. For example, normal human fibroblast membranes were less perforated by TTFields than glioma cells (16). Neurons and glia however were not completely spared as cells showed lower frequencies with 3 days of exposure. However, the degrees of changes were far less than glioma cells. For example, neuronal cilia frequencies after 3 continuous days of TTFields were reduced by ~40% compared with control whereas the ciliary frequency in glioma cells was reduced by ~90%. The preservation of cilia from this stress could be cytoprotective during TTFields therapy. Primary cilia on neurons, for example, were recently reported to prevent neurite degeneration in developing cortical neurons after exposure to stressors *in vivo* including alcohol and ketamine (62). Similarly in normal glia, it was reported that hedgehog-mediated signaling thru primary cilia promotes cell survival in stressed *in vitro* conditions (63). Thus, the retention of cilia on neurons and glia may help protect against stress induced by TTFields. The extent to which TTFields affects tumor cells and normal neural cilia in the human brain will require post-mortem analyses.

Our findings raise the possibility that TTFields could help eliminate or suppress TMZ-resistant cell types. TMZ can stimulate expression of ARL13B and an interaction between ARL13B and the purine biosynthesis pathway as a mechanism to drive TMZ chemoresistance in glioblastoma (30). Thus, TMZ stimulation of ciliogenesis that we and others found, and increased sensitivity to TMZ in ARL13B KO cells, are consistent with the notion that cilia contribute to resistance to treatment. Thus, TTFields targeting of cilia or ciliated glioma cells may enhance TMZ toxicity. Given the opposing effects on TMZ and TTFields on ciliogenesis, we explored the effects of the different steps of these treatments and found that the interaction on subsequent proliferation depends on when TMZ is administered. Surprisingly, we observed that simultaneous treatment of TTFields with TMZ worked against the efficacy of TTFields (**Figure 7G, H**), through some unknown mechanism. However, TMZ before or after TTFields application slowed tumor cell recurrence, an effect not observed in ARL13B-depleted cells. The implications may be twofold. First, the combined effects of TMZ and TTFields may depend on the degree of ARL13B^+^ cilia in the tumor. Second, there may be a treatment window or boundaries surrounding TTFields, within which TMZ exposure may be counterproductive against slowing tumor recurrence. Whether TMZ and TTFields have converging actions at the cilium remains unclear. Both treatments appear capable of stimulating autophagy (14, 64, 65), yet their effects on ciliogenesis appear opposite. Nevertheless, autophagy pathway inhibitors during or subsequent to these treatments could help target cells that clearly survive both treatments.

While there seems to be clear differences in the way TTFields and TMZ affect ciliogenesis, there is also a strong possibility that each treatment-induced effect on ciliogenesis is not directly related. There are many membrane and intracellular pathways altered within the same timeframe by TTFields that could secondarily lead to ciliary disruptions (6). Similarly, the mechanisms that promote TMZ resistance are complex (66). Many TMZ-induced DNA damage response pathways or epigenetic changes could affect cilia indirectly. In the context of either TTFields or TMZ, it is not clear if cilia play initial upstream response roles. Thus, there should be caution in interpreting ciliary changes as they may not be involved in the direct mechanism of action. However, they may reflect potential readouts of each treatment. While these are clear challenges going forward, normal mouse neural cilia do not seem to display the same degree of changes in their cilia at least in response to TTFields, which may point to important biological properties of cilia that differ between transformed and nontransformed cells.

Our study raises the possibility that gliomas with enhanced ciliogenesis potential may be more sensitive to TTFields therapy, a cellular susceptibility that may come with tradeoffs. A tumor containing more ciliated proliferating cells may be more impacted by TTFields than tumors with few ciliated cells. Considering the episodic nature of the TTFields therapy, our data raise the possibility that delayed treatment intervals could lead to unwanted recurrence of ciliated tumor cells. On the other hand, a complete or sustained ablation of cilia may generate cell offspring that are mutated or transform into other resistant cell types. Indeed, some GBMs and older glioma cell lines are or become cilia devoid (26, 67, 68). In medulloblastoma, loss of cilia mediated due to ablation of *OFD1* can lead to smoothened (SMO) inhibitor treatment resistance and formation of “persister-like” states that support tumor recurrence (69). While our study involves patient-derived cell lines and fresh patient surgical resections, a limitation of our studies is we are not yet able to examine the effects of TTFields on intracranial tumor growth in an animal model, a future direction of our studies. It is possible the changes in cilia may be less robust deep inside the brain. Nevertheless, the TTFields-mediated changes in cilia we observed *ex vivo* warrant these investigations at the organelle level. Though all available genomic or proteomic databases cannot provide any information about the degree of ciliation in a tumor, a recent study found that increased expression of ciliary genes is a predictor of poor survival in glioma (70). Future studies will need to examine how survival of patients being treated with TTFields and TMZ therapy depends on the ciliated profile of their tumors.

## Data Availability Statement

The raw data supporting the conclusions of this article will be made available by the authors, without undue reservation.

## Ethics Statement

The studies involving human participants were reviewed and approved by the University of Florida Institutional Review Board (Protocol #201902489). Written informed consent for participation was not required for this study in accordance with the national legislation and the institutional requirements.

## Author Contributions

PS, LD, and MS contributed to the conception and design of the study. PS, JT, BU, JM, and MS performed the experiments and collected and analyzed the data. BU, HK, and LD contributed key resources for the experiments. PS, JT, and MS performed the statistical analysis. PS, JT, BU, HK, LD and MS contributed to writing and editing of the manuscript. All authors contributed to manuscript revision and read and approved the submitted version.

## Funding

Funding for this research was supported in part by a grant from the Florida Center for Brain Tumor Research and Accelerate Brain Cancer Cured (#MOG06 to MS) and a 2019 American Association for Cancer Research (AACR)-Novocure Tumor Treating Fields Research Grant (#19-60-62-SARK to MS).

## Conflict of Interest

The work is in part supported by Novocure Inc. The corresponding author is a paid consultant for Novocure Inc.

## Publisher’s Note

All claims expressed in this article are solely those of the authors and do not necessarily represent those of their affiliated organizations, or those of the publisher, the editors and the reviewers. Any product that may be evaluated in this article, or claim that may be made by its manufacturer, is not guaranteed or endorsed by the publisher.

## References

[1] Stupp R Hegi ME Mason WP van den Bent MJ Taphoorn MJ Janzer RC. Effects of Radiotherapy With Concomitant and Adjuvant Temozolomide Versus Radiotherapy Alone on Survival in Glioblastoma in a Randomised Phase III Study: 5-Year Analysis of the EORTC-NCIC Trial. Lancet Oncol (2009) 10(5):459–66. doi: 10.1016/S1470-2045(09)70025-7 19269895

[2] Stupp R Mason WP van den Bent MJ Weller M Fisher B Taphoorn MJ. Radiotherapy Plus Concomitant and Adjuvant Temozolomide for Glioblastoma. N Engl J Med (2005) 352(10):987–96. doi: 10.1056/NEJMoa043330 15758009

[3] Stupp R Taillibert S Kanner A Read W Steinberg DM Lhermitte B. Effect of Tumor-Treating Fields Plus Maintenance Temozolomide vs Maintenance Temozolomide Alone on Survival in Patients With Glioblastoma: A Randomized Clinical Trial. JAMA (2017) 318(23):2306–16. doi: 10.1001/jama.2017.18718 PMC5820703 29260225

[4] Stupp R Taillibert S Kanner AA Kesari S Steinberg DM Toms SA. Maintenance Therapy With Tumor-Treating Fields Plus Temozolomide vs Temozolomide Alone for Glioblastoma: A Randomized Clinical Trial. JAMA (2015) 314(23):2535–43. doi: 10.1001/jama.2015.16669 26670971

[5] Mehta M Wen P Nishikawa R Reardon D Peters K. Critical Review of the Addition of Tumor Treating Fields (TTFields) to the Existing Standard of Care for Newly Diagnosed Glioblastoma Patients. Crit Rev Oncol Hematol (2017) 111:60–5. doi: 10.1016/j.critrevonc.2017.01.005 28259296

[6] Karanam NK Story MD. An Overview of Potential Novel Mechanisms of Action Underlying Tumor Treating Fields-Induced Cancer Cell Death and Their Clinical Implications. Int J Radiat Biol (2021) 97(8):1044–54. doi: 10.1080/09553002.2020.1837984 33086019

[7] Kirson ED Gurvich Z Schneiderman R Dekel E Itzhaki A Wasserman Y. Disruption of Cancer Cell Replication by Alternating Electric Fields. Cancer Res (2004) 64(9):3288–95. doi: 10.1158/0008-5472.CAN-04-0083 15126372

[8] Kirson ED Dbaly V Tovarys F Vymazal J Soustiel JF Itzhaki A. Alternating Electric Fields Arrest Cell Proliferation in Animal Tumor Models and Human Brain Tumors. Proc Natl Acad Sci USA (2007) 104(24):10152–7. doi: 10.1073/pnas.0702916104 PMC1886002 17551011

[9] Giladi M Schneiderman RS Voloshin T Porat Y Munster M Blat R. Mitotic Spindle Disruption by Alternating Electric Fields Leads to Improper Chromosome Segregation and Mitotic Catastrophe in Cancer Cells. Sci Rep (2015) 5:18046. doi: 10.1038/srep18046 26658786 PMC4676010

[10] Kim EH Song HS Yoo SH Yoon M. Tumor Treating Fields Inhibit Glioblastoma Cell Migration, Invasion and Angiogenesis. Oncotarget (2016) 7(40):65125–36. doi: 10.18632/oncotarget.11372 PMC5323142 27556184

[11] Silginer M Weller M Stupp R Roth P. Biological Activity of Tumor-Treating Fields in Preclinical Glioma Models. Cell Death Dis (2017) 8(4):e2753. doi: 10.1038/cddis.2017.171 28425987 PMC5477589

[12] Schneiderman RS Shmueli E Kirson ED Palti Y. TTFields Alone and in Combination With Chemotherapeutic Agents Effectively Reduce the Viability of MDR Cell Sub-Lines That Over-Express ABC Transporters. BMC Cancer (2010) 10:229. doi: 10.1186/1471-2407-10-229 20492723 PMC2893108

[13] Kessler AF Frombling GE Gross F Hahn M Dzokou W Ernestus RI. Effects of Tumor Treating Fields (TTFields) on Glioblastoma Cells Are Augmented by Mitotic Checkpoint Inhibition. Cell Death Discovery (2018) 4:12. doi: 10.1038/s41420-018-0079-9 PMC6125382 30210815

[14] Shteingauz A Porat Y Voloshin T Schneiderman RS Munster M Zeevi E. AMPK-Dependent Autophagy Upregulation Serves as a Survival Mechanism in Response to Tumor Treating Fields (TTFields). Cell Death Dis (2018) 9(11):1074. doi: 10.1038/s41419-018-1085-9 30341282 PMC6195570

[15] Voloshin T Kaynan N Davidi S Porat Y Shteingauz A Schneiderman RS. Tumor-Treating Fields (TTFields) Induce Immunogenic Cell Death Resulting in Enhanced Antitumor Efficacy When Combined With Anti-PD-1 Therapy. Cancer Immunol Immunother (2020) 69(7):1191–204. doi: 10.1007/s00262-020-02534-7 PMC7303058 32144446

[16] Chang E Patel CB Pohling C Young C Song J Flores TA. Tumor Treating Fields Increases Membrane Permeability in Glioblastoma Cells. Cell Death Discovery (2018) 4:113. doi: 10.1038/s41420-018-0130-x 30534421 PMC6281619

[17] Neuhaus E Zirjacks L Ganser K Klumpp L Schuler U Zips D. Alternating Electric Fields (TTFields) Activate Cav1.2 Channels in Human Glioblastoma Cells. Cancers (Basel) (2019) 11(1):110. doi: 10.3390/cancers11010110 PMC6356873 30669316

[18] Goetz SC Anderson KV. The Primary Cilium: A Signalling Centre During Vertebrate Development. Nat Rev Genet (2010) 11(5):331–44. doi: 10.1038/nrg2774 PMC3121168 20395968

[19] Wheway G Nazlamova L Hancock JT. Signaling Through the Primary Cilium. Front Cell Dev Biol (2018) 6:8. doi: 10.3389/fcell.2018.00008 29473038 PMC5809511

[20] Reiter JF Leroux MR. Genes and Molecular Pathways Underpinning Ciliopathies. Nat Rev (2017) 18(9):533–47. doi: 10.1038/nrm.2017.60 PMC5851292 28698599

[21] Garcia G 3rd Raleigh DR Reiter JF. How the Ciliary Membrane Is Organized Inside-Out to Communicate Outside-In. Curr Biol (2018) 28(8):R421–34. doi: 10.1016/j.cub.2018.03.010 PMC6434934 29689227

[22] Garcia-Gonzalo FR Phua SC Roberson EC Garcia G 3rd Abedin M Schurmans S. Phosphoinositides Regulate Ciliary Protein Trafficking to Modulate Hedgehog Signaling. Dev Cell (2015) 34(4):400–9. doi: 10.1016/j.devcel.2015.08.001 PMC4557815 26305592

[23] Garcia-Gonzalo FR Reiter JF. Scoring a Backstage Pass: Mechanisms of Ciliogenesis and Ciliary Access. J Cell Biol (2012) 197(6):697–709. doi: 10.1083/jcb.201111146 22689651 PMC3373398

[24] Raleigh DR Sever N Choksi PK Sigg MA Hines KM Thompson BM. Cilia-Associated Oxysterols Activate Smoothened. Mol Cell (2018) 72(2):316–27.e315. doi: 10.1016/j.molcel.2018.08.034 30340023 PMC6503851

[25] Rosenbaum JL Witman GB. Intraflagellar Transport. Nat Rev (2002) 3(11):813–25. doi: 10.1038/nrm952 12415299

[26] Sarkisian MR Siebzehnrubl D Hoang-Minh L Deleyrolle L Silver DJ Siebzehnrubl FA. Detection of Primary Cilia in Human Glioblastoma. J Neurooncol (2014) 117(1):15–24. doi: 10.1007/s11060-013-1340-y 24510433 PMC4433742

[27] Cai S Bodle JC Mathieu PS Amos A Hamouda M Bernacki S. Primary Cilia are Sensors of Electrical Field Stimulation to Induce Osteogenesis of Human Adipose-Derived Stem Cells. FASEB J (2017) 31(1):346–55. doi: 10.1096/fj.201600560r PMC5161527 27825103

[28] Chen Y Aspera-Werz RH Menger MM Falldorf K Ronniger M Stacke C. Exposure to 16 Hz Pulsed Electromagnetic Fields Protect the Structural Integrity of Primary Cilia and Associated TGF-Beta Signaling in Osteoprogenitor Cells Harmed by Cigarette Smoke. Int J Mol Sci (2021) 22(13):7036. doi: 10.3390/ijms22137036 34210094 PMC8268780

[29] Hoang-Minh L Deleyrolle L Nakamura N Parker A Martuscello R Reynolds B. PCM1 Depletion Inhibits Glioblastoma Cell Ciliogenesis and Increases Cell Death and Sensitivity to Temozolomide. Transl Oncol (2016) 9(5):392–402. doi: 10.1016/j.tranon.2016.08.006 27661404 PMC5035360

[30] Shireman JM Atashi F Lee G Ali ES Saathoff MR Park CH. *De Novo* Purine Biosynthesis Is a Major Driver of Chemoresistance in Glioblastoma. Brain (2021) 144(4):1230–46. doi: 10.1093/brain/awab020 PMC8105035 33855339

[31] Hoang-Minh LB Dutra-Clarke M Breunig JJ Sarkisian MR. Glioma Cell Proliferation Is Enhanced in the Presence of Tumor-Derived Cilia Vesicles. Cilia (2018) 7:6. doi: 10.1186/s13630-018-0060-5 30410731 PMC6219037

[32] Shi P Hoang-Minh LB Tian J Cheng A Basrai R Kalaria N. HDAC6 Signaling at Primary Cilia Promotes Proliferation and Restricts Differentiation of Glioma Cells. Cancers (Basel) (2021) 13(7):1644. doi: 10.3390/cancers13071644 33915983 PMC8036575

[33] Deleyrolle LP Harding A Cato K Siebzehnrubl FA Rahman M Azari H. Evidence for Label-Retaining Tumour-Initiating Cells in Human Glioblastoma. Brain (2011) 134(Pt 5):1331–43. doi: 10.1093/brain/awr081 PMC3097894 21515906

[34] Hothi P Martins TJ Chen L Deleyrolle L Yoon JG Reynolds B. High-Throughput Chemical Screens Identify Disulfiram as an Inhibitor of Human Glioblastoma Stem Cells. Oncotarget (2012) 3(10):1124–36. doi: 10.18632/oncotarget.707 PMC3717950 23165409

[35] Lin B Lee H Yoon JG Madan A Wayner E Tonning S. Global Analysis of H3K4me3 and H3K27me3 Profiles in Glioblastoma Stem Cells and Identification of SLC17A7 as a Bivalent Tumor Suppressor Gene. Oncotarget (2015) 6(7):5369–81. doi: 10.18632/oncotarget.3030 PMC4467155 25749033

[36] Dagra A Miller DR Lin M Gopinath A Shaerzadeh F Harris S. Alpha-Synuclein-Induced Dysregulation of Neuronal Activity Contributes to Murine Dopamine Neuron Vulnerability. NPJ Parkinsons Dis (2021) 7(1):76. doi: 10.1038/s41531-021-00210-w 34408150 PMC8373893

[37] Porat Y Giladi M Schneiderman RS Blat R Shteingauz A Zeevi E. Determining the Optimal Inhibitory Frequency for Cancerous Cells Using Tumor Treating Fields (TTFields). J Vis Exp (2017) (123):55820. doi: 10.3791/55820 PMC5607886 28518093

[38] Hoang-Minh LB Deleyrolle LP Siebzehnrubl D Ugartemendia G Futch H Griffith B. Disruption of KIF3A in Patient-Derived Glioblastoma Cells: Effects on Ciliogenesis, Hedgehog Sensitivity, and Tumorigenesis. Oncotarget (2016) 7:7029–43. doi: 10.18632/oncotarget.6854 PMC4872766 26760767

[39] Linder B Schiesl A Voss M Rodel F Hehlgans S Gullulu O. Dexamethasone Treatment Limits Efficacy of Radiation, But Does Not Interfere With Glioma Cell Death Induced by Tumor Treating Fields. Front Oncol (2021) 11:715031. doi: 10.3389/fonc.2021.715031 34395289 PMC8361446

[40] Romio L Fry AM Winyard PJ Malcolm S Woolf AS Feather SA. OFD1 Is a Centrosomal/Basal Body Protein Expressed During Mesenchymal-Epithelial Transition in Human Nephrogenesis. J Am Soc Nephrol (2004) 15(10):2556–68. doi: 10.1097/01.ASN.0000140220.46477.5C 15466260

[41] Singla V Romaguera-Ros M Garcia-Verdugo JM Reiter JF. Ofd1, a Human Disease Gene, Regulates the Length and Distal Structure of Centrioles. Dev Cell (2010) 18(3):410–24. doi: 10.1016/j.devcel.2009.12.022 PMC2841064 20230748

[42] Tang Z Lin MG Stowe TR Chen S Zhu M Stearns T. Autophagy Promotes Primary Ciliogenesis by Removing OFD1 From Centriolar Satellites. Nature (2014) 502(7470):254–7. doi: 10.1038/505254a PMC4075283 24089205

[43] Qiu H Fujisawa S Nozaki S Katoh Y Nakayama K. Interaction of INPP5E With ARL13B Is Essential for Its Ciliary Membrane Retention But Dispensable for Its Ciliary Entry. Biol Open (2021) 10(1):bio057653. doi: 10.1242/bio.057653 33372066 PMC7860134

[44] Arellano JI Guadiana SM Breunig JJ Rakic P Sarkisian MR. Development and Distribution of Neuronal Cilia in Mouse Neocortex. J Comp Neurol (2012) 520(4):848–73. doi: 10.1002/cne.22793 PMC3325766 22020803

[45] Berbari NF Bishop GA Askwith CC Lewis JS Mykytyn K. Hippocampal Neurons Possess Primary Cilia in Culture. J Neurosci Res (2007) 85(5):1095–100. doi: 10.1002/jnr.21209 17304575

[46] Bishop GA Berbari NF Lewis J Mykytyn K. Type III Adenylyl Cyclase Localizes to Primary Cilia Throughout the Adult Mouse Brain. J Comp Neurol (2007) 505(5):562–71. doi: 10.1002/cne.21510 17924533

[47] Guadiana SM Semple-Rowland SL Daroszewski D Madorsky I Breunig JJ Mykytyn K. Arborization of Dendrites by Developing Neocortical Neurons is Dependent on Primary Cilia and Type 3 Adenylyl Cyclase. J Neurosci (2013) 33:2626–38. doi: 10.1523/JNEUROSCI.2906-12.2013 PMC6619186 23392690

[48] Parker AK Le MM Smith TS Hoang-Minh LB Atkinson EW Ugartemendia G. Neonatal Seizures Induced by Pentylenetetrazol or Kainic Acid Disrupt Primary Cilia Growth on Developing Mouse Cortical Neurons. Exp Neurol (2016) 282:119–27. doi: 10.1016/j.expneurol.2016.05.015 27181411

[49] Wang L Dynlacht BD. The Regulation of Cilium Assembly and Disassembly in Development and Disease. Development (2018) 145(18):dev151407. doi: 10.1242/dev.151407 30224385 PMC6176931

[50] Praetorius HA Spring KR. Bending the MDCK Cell Primary Cilium Increases Intracellular Calcium. J Membr Biol (2001) 184(1):71–9. doi: 10.1007/s00232-001-0075-4 11687880

[51] Peixoto E Jin S Thelen K Biswas A Richard S Morleo M. HDAC6-Dependent Ciliophagy Is Involved in Ciliary Loss and Cholangiocarcinoma Growth in Human Cells and Murine Models. Am J Physiol Gastrointest Liver Physiol (2020) 318(6):G1022–33. doi: 10.1152/ajpgi.00033.2020 PMC7311663 32338033

[52] Akhshi T Trimble WS. A non-Canonical Hedgehog Pathway Initiates Ciliogenesis and Autophagy. J Cell Biol (2021) 220(1):e202004179. doi: 10.1083/jcb.202004179 33258871 PMC7714386

[53] Magistrati E Maestrini G Nino CA Lince-Faria M Beznoussenko G Mironov A. Myosin VI Regulates Ciliogenesis by Promoting the Turnover of the Centrosomal/Satellite Protein OFD1. EMBO Rep (2021) e54160. doi: 10.1101/2021.06.18.448975 34957672 PMC8892233

[54] Morleo M Brillante S Formisano U Ferrante L Carbone F Iaconis D. Regulation of Autophagosome Biogenesis by OFD1-Mediated Selective Autophagy. EMBO J (2021) 40(4):e105120. doi: 10.15252/embj.2020105120 33368531 PMC7883294

[55] Pampliega O Orhon I Patel B Sridhar S Diaz-Carretero A Beau I. Functional Interaction Between Autophagy and Ciliogenesis. Nature (2014) 502(7470):194–200. doi: 10.1038/nature12639 PMC3896125 24089209

[56] Hashimoto M Tanaka H Oguro K Masuzawa T. Subependymoma of the Lateral Ventricle–Case Report. Neurol Med Chir (Tokyo) (1991) 31(11):732–5. doi: 10.2176/nmc.31.732 1723163

[57] Das RM Storey KG. Apical Abscission Alters Cell Polarity and Dismantles the Primary Cilium During Neurogenesis. Science (2014) 343(6167):200–4. doi: 10.1126/science.1247521 PMC4066580 24408437

[58] Mirvis M Siemers KA Nelson WJ Stearns TP. Primary Cilium Loss in Mammalian Cells Occurs Predominantly by Whole-Cilium Shedding. PloS Biol (2019) 17(7):e3000381. doi: 10.1371/journal.pbio.3000381 31314751 PMC6699714

[59] Ford MJ Yeyati PL Mali GR Keighren MA Waddell SH Mjoseng HK. A Cell/Cilia Cycle Biosensor for Single-Cell Kinetics Reveals Persistence of Cilia After G1/S Transition is a General Property in Cells and Mice. Dev Cell (2018) 47(4):509–23.e505. doi: 10.1016/j.devcel.2018.10.027 30458140 PMC6251972

[60] Wang S Livingston MJ Su Y Dong Z. Reciprocal Regulation of Cilia and Autophagy *via* the MTOR and Proteasome Pathways. Autophagy (2015) 11(4):607–16. doi: 10.1080/15548627.2015.1023983 PMC4502771 25906314

[61] Lam HC Cloonan SM Bhashyam AR Haspel JA Singh A Sathirapongsasuti JF. Histone Deacetylase 6-Mediated Selective Autophagy Regulates COPD-Associated Cilia Dysfunction. J Clin Invest (2013) 123(12):5212–30. doi: 10.1172/JCI69636 PMC3859407 24200693

[62] Ishii S Sasaki T Mohammad S Hwang H Tomy E Somaa F. Primary Cilia Safeguard Cortical Neurons in Neonatal Mouse Forebrain From Environmental Stress-Induced Dendritic Degeneration. Proc Natl Acad Sci USA (2021) 118(1):e2012482118. doi: 10.1073/pnas.2012482118 33443207 PMC7817201

[63] Yoshimura K Kawate T Takeda S. Signaling Through the Primary Cilium Affects Glial Cell Survival Under a Stressed Environment. Glia (2011) 59(2):333–44. doi: 10.1002/glia.21105 21125655

[64] Wurstle S Schneider F Ringel F Gempt J Lammer F Delbridge C. Temozolomide Induces Autophagy in Primary and Established Glioblastoma Cells in an EGFR Independent Manner. Oncol Lett (2017) 14(1):322–8. doi: 10.3892/ol.2017.6107 PMC5494811 28693171

[65] Yan Y Xu Z Dai S Qian L Sun L Gong Z. Targeting Autophagy to Sensitive Glioma to Temozolomide Treatment. J Exp Clin Cancer Res (2016) 35:23. doi: 10.1186/s13046-016-0303-5 26830677 PMC4736617

[66] Lee SY. Temozolomide Resistance in Glioblastoma Multiforme. Genes Dis (2016) 3(3):198–210. doi: 10.1016/j.gendis.2016.04.007 30258889 PMC6150109

[67] Moser JJ Fritzler MJ Rattner JB. Primary Ciliogenesis Defects Are Associated With Human Astrocytoma/Glioblastoma Cells. BMC Cancer (2009) 9:448. doi: 10.1186/1471-2407-9-448 20017937 PMC2806408

[68] Moser JJ Fritzler MJ Rattner JB. Ultrastructural Characterization of Primary Cilia in Pathologically Characterized Human Glioblastoma Multiforme (GBM) Tumors. BMC Clin Pathol (2014) 14:40. doi: 10.1186/1472-6890-14-40 25228849 PMC4164667

[69] Zhao X Pak E Ornell KJ Pazyra-Murphy MF MacKenzie EL Chadwick EJ. A Transposon Screen Identifies Loss of Primary Cilia as a Mechanism of Resistance to SMO Inhibitors. Cancer Discovery (2017) 7(12):1436–49. doi: 10.1158/2159-8290.CD-17-0281 28923910

[70] Rajagopalan S Singh A Khiabanian H. Cilium Expression Score Predicts Glioma Survival. Front Genet (2021) 12:758391. doi: 10.3389/fgene.2021.758391 34868236 PMC8640099

